# HIF-2α drives osteoarthritis progression via suppression of the HDAC4-ATF4-CHOP signaling axis

**DOI:** 10.1371/journal.pone.0351847

**Published:** 2026-06-18

**Authors:** Pinpin Jiang, Hang Wang, Yujia Li, Yuanyu Zhang, Jingrui Huang, Yukun Xu, Dahai Rong, Danni Ruan, Yao Wang, Jie Yuan, Pengcui Li

**Affiliations:** 1 Key Laboratory of Bone and Soft Tissue Injury, Second Hospital of Shanxi Medical University, Taiyuan, China; 2 Academy of Medical Sciences, Shanxi Medical University, Taiyuan, China; West Virginia University School of Medicine, UNITED STATES OF AMERICA

## Abstract

Hypoxia-inducible factor-2α (HIF-2α) is a key regulator of cellular adaptation to hypoxia, but its role in osteoarthritis (OA) remains incompletely defined. This study aimed to investigate the contribution of HIF-2α to OA pathogenesis and the underlying molecular mechanisms. Human cartilage specimens were collected to examine HIF-2α expression and components of the histone deacetylase 4 (HDAC4) signaling pathway using Western blotting, quantitative real-time PCR, and immunohistochemistry. An *in vitro* OA model was established in chondrocytes using interleukin-1β (IL-1β), followed by HIF-2α knockdown with small interfering RNA and overexpression via adenoviral transduction. Chondrocyte apoptosis was assessed by flow cytometry and TUNEL staining. To evaluate *in vivo* effects, HIF-2α was silenced using an adeno-associated viral vector in a rat OA model induced by anterior cruciate ligament transection (ACLT). Disease progression was assessed by X-ray, computed tomography (CT), FMT® small animal *in vivo* fluorescence molecular tomography imaging system, Safranin O staining, and immunohistochemistry. HIF-2α expression was significantly increased in cartilage from OA patients and ACLT rats. *In vitro*, HIF-2α modulation altered HDAC4 expression and downstream apoptotic signaling. Knockdown of HIF-2α reduced chondrocyte apoptosis and attenuated cartilage degeneration *in vivo*. These findings indicate that HIF-2α promotes OA progression by regulating chondrocyte apoptosis and matrix homeostasis through the HDAC4-ATF4-CHOP pathway. This study identifies a previously unrecognized mechanism linking HIF-2α to OA and highlights its potential as a therapeutic target.

## 1. Introduction

Osteoarthritis (OA) is a common degenerative joint disease associated with aging, and its prevalence continues to rise as the population ages [1]. Despite its high burden, effective disease-modifying treatments remain limited, and joint replacement is often the only option for patients with advanced disease. Articular cartilage is an avascular and aneural tissue that relies on diffusion from synovial fluid and the subchondral bone for nutrients and oxygen. As a result, chondrocytes exist in a relatively hypoxic microenvironment under physiological conditions [2].

Hypoxia-inducible factors (HIFs) are key transcriptional regulators of cellular adaptation to low oxygen tension and play important roles in cartilage homeostasis and OA progression [3,4]. Among them, hypoxia-inducible factor-2α (HIF-2α) has emerged as an important mediator of chondrocyte responses to hypoxia. HIF-2α regulates the expression of a range of target genes involved in processes such as apoptosis, angiogenesis, and cellular differentiation, and is critical for maintaining chondrocyte function and phenotype [5,6].

Histone deacetylase 4 (HDAC4) is a key regulator of cartilage development and homeostasis. It suppresses chondrocyte hypertrophy and endochondral ossification, thereby preserving cartilage integrity [7–9]. In addition, HDAC4 influences cell proliferation and apoptosis through transcriptional and post-transcriptional regulation [10]. Notably, HDAC4 expression has been reported to decline progressively in OA cartilage [11]. Reduced HDAC4 activity may relieve its inhibitory effect on activating transcription factor 4 (ATF4), leading to activation of the ATF4/CHOP signaling pathway, which has been implicated in chondrocyte apoptosis [12,13]. These findings suggest that restoring HDAC4 function or targeting the ATF4/CHOP axis may represent a potential therapeutic strategy for OA.

In this study, we sought to define the role of HIF-2α in OA and to elucidate the underlying molecular mechanisms. We first examined HIF-2α expression in human OA cartilage and in experimental OA models. We then investigated its functional effects on cartilage degeneration and chondrocyte apoptosis, with a focus on the HDAC4-ATF4-CHOP signaling pathway. Our findings identify HIF-2α as a regulator of this axis and provide new insight into its contribution to OA pathogenesis, highlighting its potential as a therapeutic target.

## 2. Methods

### 2.1 Human cartilage samples

This study was approved by the Ethics Committee of the Second Hospital of Shanxi Medical University (Taiyuan, China; approval no. 2022YX167). Human articular cartilage specimens were obtained from patients with osteoarthritis undergoing total knee arthroplasty between September 2022 and March 2023. Clinical characteristics of the patients are provided in S1 Table in S1 File. Cartilage samples were classified according to the International Cartilage Regeneration & Joint Preservation Society (ICRS) grading system. Severely degenerated regions (ICRS grades 3–4) were defined as the OA group, whereas macroscopically preserved regions (ICRS grades 0–1) from the same joints served as the relatively normal group. All procedures were conducted in accordance with approved protocols, and written informed consent was obtained from all participants.

### 2.2 Cell culture and treatment

The human chondrocyte cell line C28/I2 (iCell Bioscience Inc.) was cultured in high-glucose medium supplemented with 10% fetal bovine serum and 1% penicillin-streptomycin. Cells were assigned to the following groups: Blank, IL-1β, HIF-2α overexpression (HIF-2α-OE), adenoviral negative control (Ad-NC), HIF-2α siRNA (si-HIF-2α), and control siRNA. For OA induction, cells at 80−90% confluence were treated with interleukin-1β (IL-1β; 20 ng/mL) for 16 hours [14]. Control cells received an equal volume of saline. For overexpression, cells were transduced with an adenoviral vector carrying HIF-2α; the medium was replaced after 8 hours, and transduction efficiency was confirmed by fluorescence microscopy at 24 hours. IL-1β was added 48 hours post-transduction, followed by a further 16-hour incubation. The Ad-NC group was treated with a control adenovirus under the same conditions. For knockdown experiments, cells were transfected with HIF-2α siRNA using Lipofectamine 3000 (Thermo Fisher Scientific) according to the manufacturer’s instructions. After 24 hours, IL-1β was added and cells were incubated for an additional 16 hours. Control siRNA was used as a negative control.

### 2.3 Westen blot analysis

Total protein was extracted using RIPA lysis buffer supplemented with PMSF. Protein samples were separated by SDS-polyacrylamide gel electrophoresis and transferred to membranes for immunoblotting. The following primary antibodies were used: anti-HIF-2α (1:1000, cat. no. ab109616, Abcam), anti-HDAC4 (1:1000, cat. no. ab271355, Abcam), anti- ATF4 (1:1000, cat. no. bs1351R, Bioss), anti-CHOP (1:1000, cat. no. bs1361R, Bioss), anti-Cleaved Caspase3 (1:1000, cat. no. bs0081R, Bioss), anti-Caspase9 (1:1000, cat. no. bs20773R, Bioss), anti-β-actin (1:1000, cat. no. ab8227, Abcam). Protein bands were visualized using a gel imaging system.

### 2.4 Quantitative real-time PCR (RT-qPCR)

Total RNA was isolated using TRIzol reagent according to the manufacturer’s protocol. Reverse transcription was performed using PrimeScript™ RT Master Mix (Takara). Quantitative PCR was conducted using TB Green Premix Ex Taq™ II (Takara) under standard cycling conditions: 95°C for 30 s, followed by 40 cycles of 95°C for 15 s and 60°C for 45 s. Primer sequences are listed in S2 Table in S1 File.

### 2.5 Flow cytometry

Apoptosis was assessed using an Annexin V-PE/7-AAD Apoptosis Detection Kit (CA1030, Solarbio, Beijing, China) according to the manufacturer’s instructions. Briefly, cells were harvested by trypsinization and resuspended in binding buffer. Cells were incubated with 5 μL Annexin V-PE for 5 minutes at room temperature in the dark, followed by the addition of 10 μL 7-AAD (20 μg/mL) and 400 μL PBS. Samples were analyzed immediately by flow cytometry.

### 2.6 TUNEL staining

Apoptotic cells were detected using a one-step TUNEL Apoptosis Detection Kit (C1089, Beyotime, Shanghai, China) following the manufacturer’s protocol. Briefly, cells were fixed in 4% paraformaldehyde and permeabilized with 0.1% Triton X-100 for 20 minutes. Cells were then incubated with TUNEL reaction solution for 2 hours at 37°C in the dark. Fluorescent signals were visualized and imaged using a fluorescence microscope.

### 2.7 Animal model

Sprague-Dawley (SD) rats were obtained from Shanxi Medical University. All animal procedures were approved by the Ethics Committee of the Second Hospital of Shanxi Medical University (approval no. DW2022063) and conducted in accordance with the NIH Guide for the Care and Use of Laboratory Animals. The study was reported in compliance with ARRIVE guidelines.

Rats were anesthetized with pentobarbital sodium, and all efforts were made to minimize discomfort. An OA model was established in the left knee by anterior cruciate ligament transection (ACLT) as previously described [15]. Animals were randomly assigned to three groups (n = 9 per group): Sham, AAV9-HIF-2α, and AAV9-HIF-2α-NC. Evaluations were performed at 4, 8, and 12 weeks after intervention. At each time point, animals were euthanized by CO_2_ inhalation in accordance with institutional guidelines.

### 2.8 Intra-articular injection of adeno-associated virus (AAV)

An adeno-associated virus targeting HIF-2α (AAV9-Epas1-RNAi; Gikai Genetics) was used for *in vivo* knockdown. Two weeks after ACLT surgery, rats in the AAV9-HIF-2α and AAV9-HIF-2α-NC groups received intra-articular injections (25 μL; 1.0 × 10^13^ viral genomes/mL) into the knee joint cavity. Sham-operated rats underwent joint puncture without virus injection. The duration and conditions of the procedure were kept consistent across all groups.

### 2.9 Radiographic and imaging analysis

At 4, 8, and 12 weeks after injection, three rats from each group were randomly selected for imaging analysis. Animals were anesthetized with pentobarbital sodium and subjected to X-ray and micro-computed tomography (CT) scanning.

For fluorescence molecular tomography (FMT), rats were anesthetized and injected intra-articularly in the right knee with 10 μL ProSense 750 FAST probe. After 16 hours, animals were re-anesthetized and imaged using an *in vivo* FMT system. Following imaging, knee joints were harvested. Gross cartilage damage was assessed by India ink staining. Specimens were then fixed in 4% paraformaldehyde for 48 hours, rinsed under running water for 12 hours, decalcified in 10% EDTA for 6 weeks, and embedded in paraffin for subsequent histological analysis.

### 2.10 Gait analysis

Gait was assessed using the CatWalk XT system. At 4, 8, and 12 weeks after intra-articular injection, three rats from each group (Sham, AAV9-HIF-2α, and AAV9-HIF-2α-NC; n = 9 total) were randomly selected for analysis. Each rat was allowed to traverse the runway (80 cm × 20 cm) three times per session, and the mean of the three trials was used for analysis. Experiments were conducted in a dark, quiet environment to minimize external stimuli.

### 2.11 Immunohistochemistry

Immunohistochemical staining was performed using a non-biotin two-step method. Paraffin sections were deparaffinized, rehydrated, treated to block endogenous peroxidase activity, and subjected to antigen retrieval. Sections were incubated with primary antibodies against HIF-2α (1:200), HDAC4 (1:200), ATF4 (1:100), CHOP (1:100), cleaved caspase-3 (1:100), and caspase-9 (1:100). After incubation with a reaction enhancement solution and a polymer-based secondary antibody (goat anti-rabbit IgG), signals were visualized using DAB. Sections were counterstained with hematoxylin, differentiated, dehydrated, cleared, and mounted. Stained sections were scanned and analyzed for marker expression.

### 2.12 Toluidine blue and Saffron O staining

Cartilage specimens were fixed and stained with toluidine blue (G3660, Solarbio) and Safranin O (G1067, Solarbio) following standard protocols. Sections were mounted with neutral resin and imaged for histological evaluation.

### 2.13 FMT

FMT was used to assess matrix metalloproteinase (MMP) activity in rat knee joints at 4, 8, and 12 weeks after ACLT. The MMP-sensitive probe MMPSense 750 FAST (20 μL, 0.8 nmol; PerkinElmer) was administered via intra-articular injection 24 hours prior to imaging. Signal intensity within the knee joint was quantified using a region-of-interest (ROI) approach.

### 2.14 Immunofluorescence staining

Cells were fixed and permeabilized with 1% Triton X-100 for 60 minutes, followed by blocking with immunostaining buffer. Cells were then incubated with anti-COL2 antibody (1:100), counterstained with DAPI, and visualized under a fluorescence microscope.

### 2.15 Statistical analysis

Data are presented as mean ± standard deviation (SD). Statistical analyses were performed using SPSS and GraphPad Prism. Comparisons between two groups were conducted using the independent samples t-test, while multiple group comparisons were analyzed by one-way ANOVA followed by Tukey’s post hoc test. All experiments were performed at least three times. A *P* value < 0.05 was considered statistically significant.

## 3. Results

### 3.1 Increased HIF-2α expression, reduced HDAC4 expression, and enhanced chondrocyte apoptosis in OA tissues

**Fig 1:**
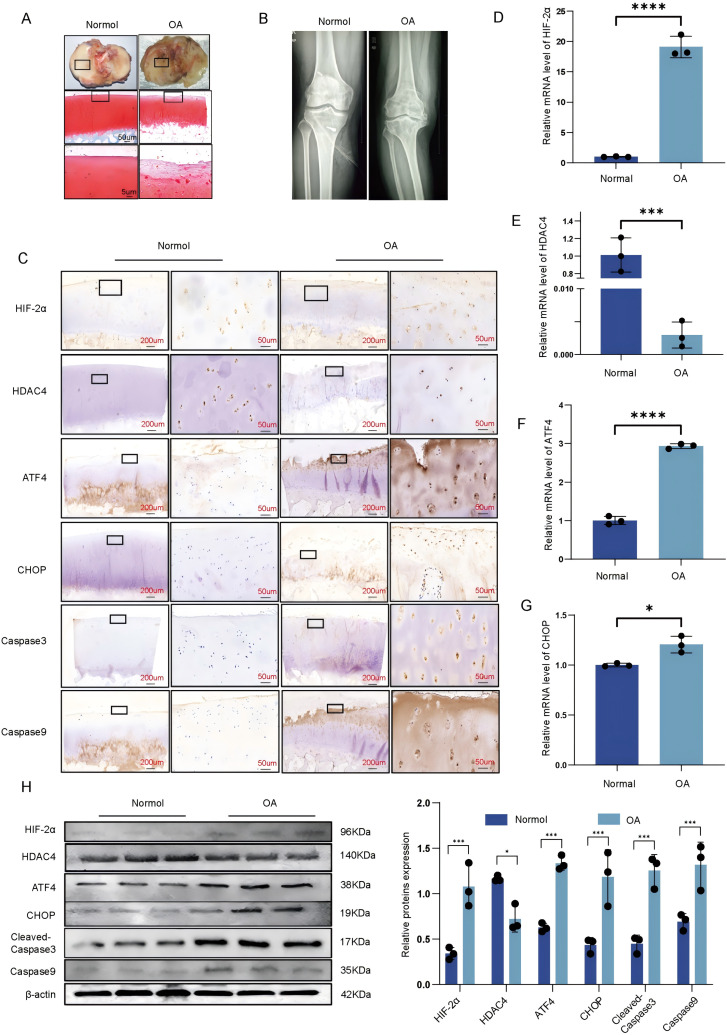
Histological and molecular changes in human cartilage. **(A)** Safranin O-fast green staining of relatively normal and OA cartilage. **(B)** Radiographic images of normal and OA knees. **(C)** Immunohistochemical staining of HDAC4, ATF4, CHOP, cleaved caspase-3, and caspase-9. **(D-G)** mRNA expression levels of HDAC4, ATF4, and CHOP. **(H)** Protein expression levels of HDAC4, ATF4, CHOP, caspase-3, and caspase-9. *P < 0.05, **P < 0.01, ***P < 0.001.

Safranin O-fast green staining was performed to evaluate histological changes in human cartilage. In OA samples, the articular cartilage surface was severely disrupted, with focal defects, thinning of the cartilage layer, reduced chondrocyte number, and formation of chondrocyte clusters. Staining intensity was markedly reduced and uneven, indicating a substantial loss of cartilage matrix proteoglycans (Fig 1A). Radiographic analysis further confirmed structural deterioration in OA knees. Compared with relatively normal joints, OA patients exhibited obvious narrowing or even disappearance of the joint space, degradation of articular cartilage, sharpening of the intercondylar eminence, and formation of marginal osteophytes (Fig 1B). At the molecular level, Western blotting and immunohistochemistry revealed significantly increased expression of HIF-2α, ATF4, CHOP, cleaved caspase-3, and caspase-9, alongside markedly reduced HDAC4 expression in OA cartilage compared with relatively normal tissue (Fig 1C and 1H). Consistently, RT-qPCR analysis showed elevated mRNA levels of HIF-2α, ATF4, and CHOP, whereas HDAC4 expression was decreased in OA samples (Fig 1D–1G).

### 3.2 HIF-2α overexpression activates the ATF4/CHOP pathway by suppressing HDAC4 and promotes chondrocyte apoptosis

**Fig 2:**
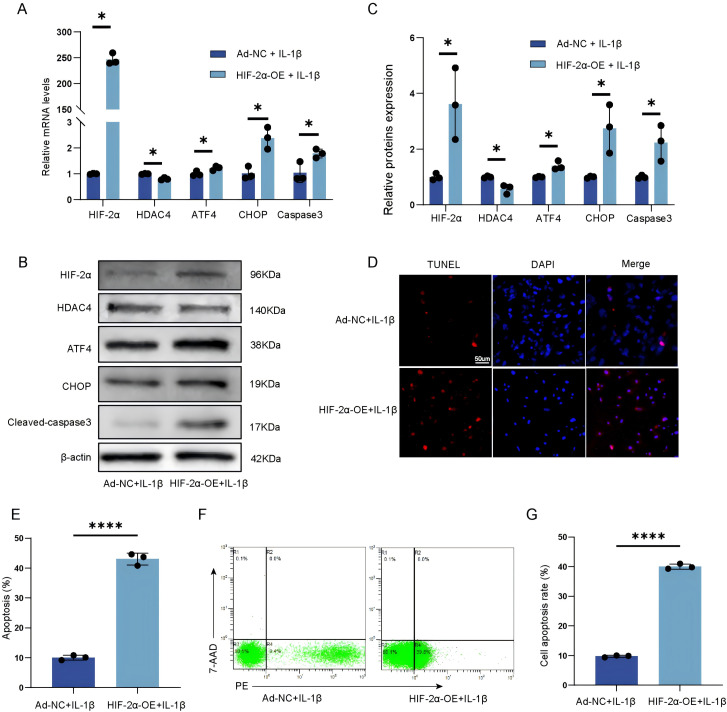
HIF-2α promotes chondrocyte apoptosis via the HDAC4-ATF4-CHOP axis. **(A)** mRNA expression of HDAC4, ATF4, CHOP, and caspase-3 after HIF-2α overexpression. **(B, C)** Protein expression of HDAC4, ATF4, and CHOP. **(D, E)** TUNEL staining showing chondrocyte apoptosis. **(F, G)** Flow cytometric analysis of apoptotic cells. *P < 0.05, **P < 0.01, ***P < 0.001.

Given the elevated expression of HIF-2α observed in OA tissues, we next investigated its functional role in IL-1β-induced chondrocytes. C28/I2 cells were transduced with an adenoviral vector to overexpress HIF-2α, and the expression of HDAC4, ATF4, CHOP, and apoptosis-related markers was assessed. HIF-2α overexpression significantly reduced HDAC4 expression while increasing the expression of ATF4 and CHOP. Meanwhile, cleaved caspase-3 levels were markedly elevated, indicating enhanced apoptotic signaling (Fig 2B and 2C). RT-qPCR results showed a consistent pattern at the mRNA level (Fig 2A). Functionally, TUNEL staining demonstrated a significant increase in apoptotic chondrocytes following HIF-2α overexpression (Fig 2D and 2E). Flow cytometry further confirmed an increased proportion of apoptotic cells in the HIF-2α overexpression group compared with controls (Fig 2F and 2G).

### 3.3 Inhibition of HIF-2α reduces chondrocyte apoptosis by upregulating HDAC4 and suppressing the ATF4/CHOP pathway

**Fig 3:**
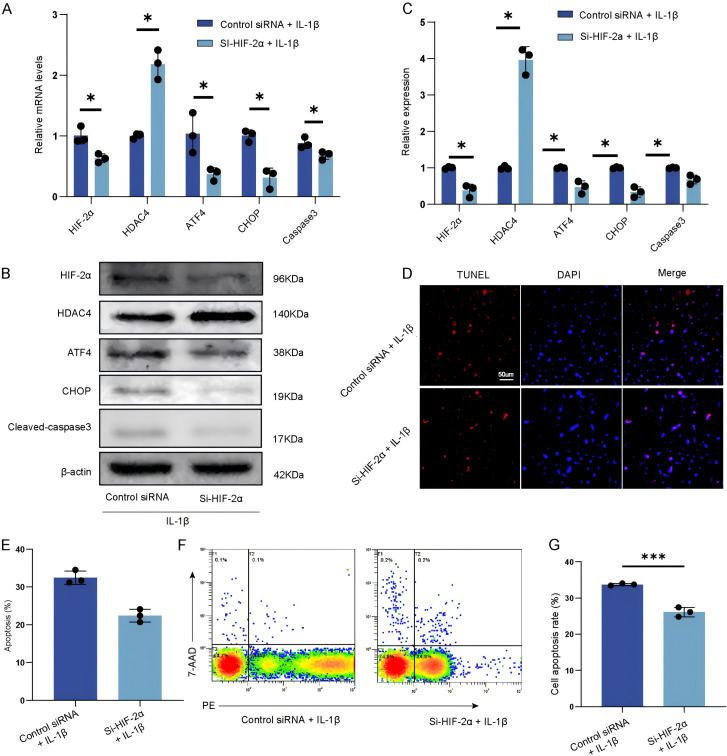
HIF-2α knockdown suppresses the ATF4/CHOP pathway and reduces chondrocyte apoptosis. **(A)** mRNA expression of HDAC4, ATF4, CHOP, and caspase-3 after HIF-2α silencing. **(B, C)** Protein expression levels of HDAC4, ATF4, CHOP, and caspase-3. **(D, E)** TUNEL staining showing chondrocyte apoptosis. **(F, G)** Flow cytometric analysis of apoptosis. *P < 0.05, **P < 0.01, ***P < 0.001.

RT-qPCR analysis showed that knockdown of HIF-2α significantly increased HDAC4 mRNA expression, while reducing the expression of ATF4 and CHOP, as well as the apoptosis-related marker caspase-3 ‌‌(Fig 3A). Consistent with the transcriptional results, Western blot analysis demonstrated a similar trend at the protein level (Fig 3B and 3C). Functionally, TUNEL staining revealed a marked reduction in chondrocyte apoptosis in the si-HIF-2α group compared with the control siRNA group (Fig 3D and 3E). Flow cytometry further confirmed a significantly lower apoptotic rate following HIF-2α knockdown (Fig 3F and 3G).

### 3.4 *In vivo* inhibition of HIF-2α promotes HDAC4 expression and suppresses the ATF4/CHOP axis

**Fig 4:**
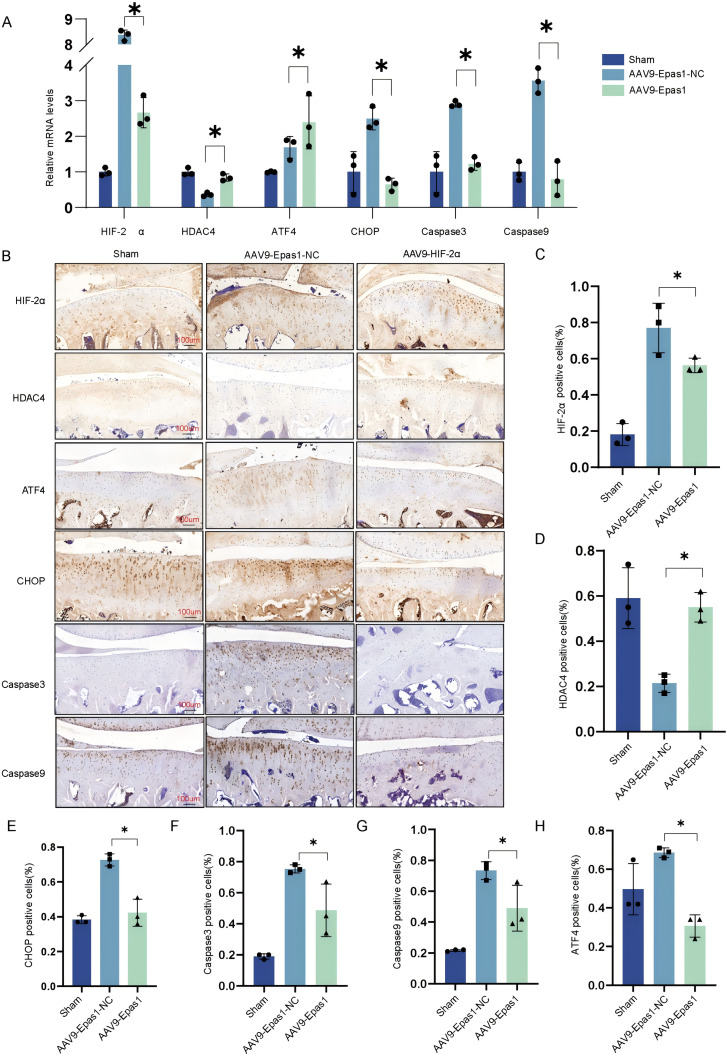
*In vivo* effects of HIF-2α knockdown on cartilage degeneration and apoptosis-related signaling. **(A)** mRNA expression of HIF-2α, HDAC4, ATF4, CHOP, caspase-3, and caspase-9 at 12 weeks. **(B-H)** Representative immunohistochemical staining and quantification of target proteins across groups. *P < 0.05, **P < 0.01, ***P < 0.001.

To further evaluate the effects of HIF-2α inhibition *in vivo*, an adeno-associated viral vector carrying HIF-2α siRNA was administered via intra-articular injection in an OA rat model. Histological analysis using H&E staining showed that the Sham group maintained an intact and smooth articular surface. In contrast, the AAV9-HIF-2α-NC group exhibited severe cartilage degeneration, including marked loss of extracellular matrix, thinning of the cartilage layer, and pronounced surface erosion. These degenerative changes were significantly alleviated in the AAV9-HIF-2α-siRNA group (Fig S1A in S1 File). Immunohistochemistry further demonstrated that, compared with the AAV9-HIF-2α-NC group, the AAV9-HIF-2α-siRNA group showed increased COL-II expression and reduced MMP13 expression (Fig S1B in S1 File), indicating improved cartilage matrix integrity. RT-qPCR analysis at 12 weeks post-surgery revealed that HIF-2α knockdown significantly decreased the expression of HIF-2α, ATF4, CHOP, caspase-3, and caspase-9, while increasing HDAC4 expression (Fig 4A). Immunohistochemical staining showed a progressive increase in HIF-2α expression over time in the OA model. However, expression levels were markedly reduced in the AAV9-HIF-2α-siRNA group compared with the AAV9-HIF-2α-NC group. At 12 weeks, strong positive staining was observed in the deep and superficial zones of cartilage in the NC group, whereas staining was significantly weaker in the siRNA-treated group. In contrast, HDAC4 expression was significantly higher in the AAV9-HIF-2α-siRNA group. Meanwhile, ATF4, CHOP, caspase-3, and caspase-9 levels were markedly reduced and approached those observed in the Sham group (Fig 4B–4H).

### 3.5 Inhibition of HIF-2α improves gait performance and reduces pain-related behavior in OA rats

**Fig 5:**
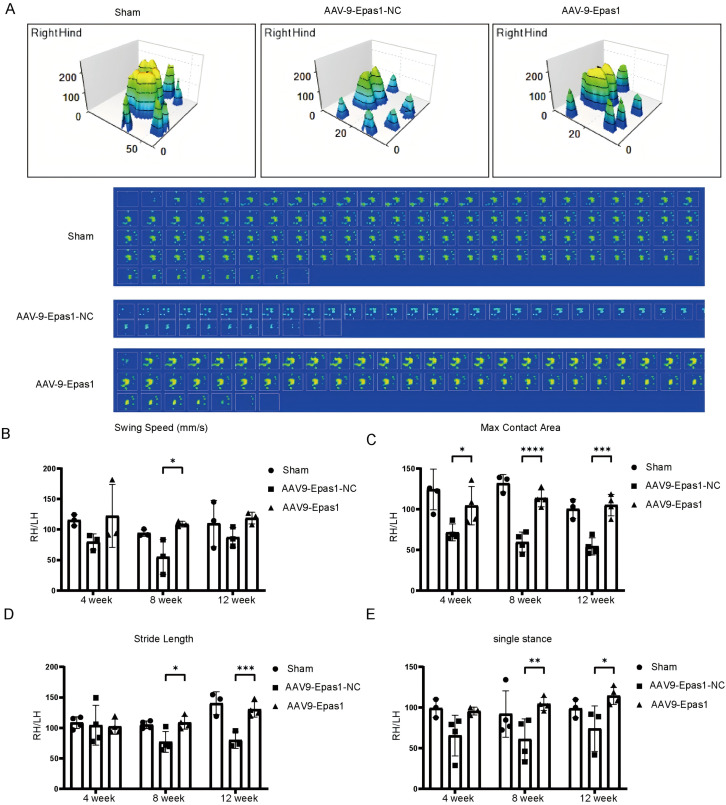
Gait analysis in Sham, AAV9-HIF-2α, and AAV9-HIF-2α-NC groups. **(A)** 3D/2D plantar pressure maps and fluorescence-based gait images. **(B-E)** Quantitative analysis of maximum contact area, stride length, single stance time, and swing speed. *P < 0.05, **P < 0.01, ***P < 0.001.

Rats from the Sham, AAV9-HIF-2α, and AAV9-HIF-2α-NC groups were randomly selected and subjected to gait analysis using the CatWalk XT system at 4, 8, and 12 weeks after intra-articular injection. Nine animals were consistently followed across time points to minimize inter-individual variability. Six gait parameters were analyzed, including body speed, maximum contact area, single stance duration, step cycle, stride length, and swing speed. Representative 3D and 2D gait maps demonstrated increased plantar pressure and prolonged paw contact time in the AAV9-HIF-2α group compared with the AAV9-HIF-2α-NC group (Fig 5A). Corresponding fluorescence intensity images also indicated greater paw contact area in the HIF-2α overexpression group (Fig 5B).

Analysis of right-to-left hind limb (RH/LH) ratios showed consistent improvements in gait parameters in the AAV9-HIF-2α group compared with controls. The maximum contact area was significantly increased at 4, 8, and 12 weeks, indicating greater weight-bearing on the affected limb (Fig 5B). Stride length was also significantly increased at 8 and 12 weeks (Fig 5C). In addition, single stance duration was prolonged at 8 and 12 weeks, suggesting improved limb support during locomotion (Fig 5D). Similarly, swing speed was significantly higher at 8 and 12 weeks in the AAV9-HIF-2α group (Fig 5E). Together, these findings indicate a progressive improvement in gait function following HIF-2α inhibition.

### 3.6 Inhibition of HIF-2α attenuates inflammatory activity and delays cartilage degeneration in OA rats

**Fig 6:**
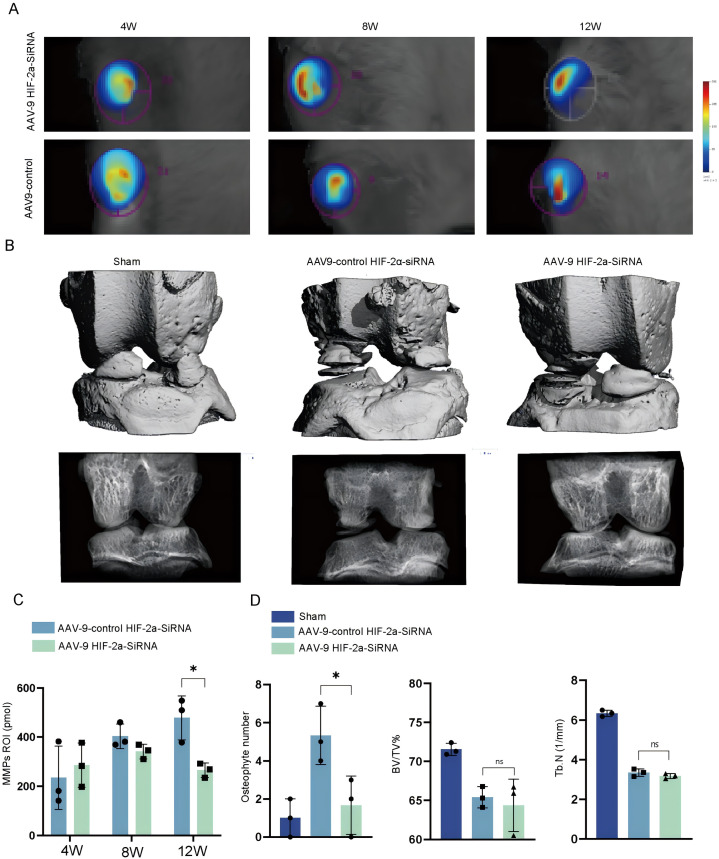
Effects of HIF-2α inhibition on inflammatory activity and joint structure in OA rats. **(A)** FMT imaging at 4, 8, and 12 weeks. **(B)** CT reconstruction and radiographic analysis. **(C)** Quantification of ROI fluorescence signals. **(D)** Quantitative analysis of bone parameters (BV/TV, Tb.N, etc.). *P < 0.05.

FMT using the ProSense 750 FAST probe was employed to evaluate MMP activity *in* *vivo*. Compared with the AAV9-HIF-2α-NC group, the AAV9-HIF-2α group showed a decreasing trend in MMP activity at 8 weeks, which became significantly lower by 12 weeks. Quantitative ROI analysis confirmed a marked reduction in fluorescence signal intensity following HIF-2α inhibition, indicating suppression of inflammatory activity in OA joints (Fig 6A and 6C). Micro-CT three-dimensional reconstruction revealed progressive joint space narrowing and increased marginal osteophyte formation in the AAV9-HIF-2α-NC group. In contrast, these degenerative changes were attenuated in the HIF-2α-inhibited group. However, no significant differences were observed in BV/TV or trabecular thickness (Tb.Th), suggesting that more pronounced alterations in subchondral bone microarchitecture may require a longer observation period to become evident (Fig 6B and 6D).

## 4. Discussion

Chondrocyte apoptosis is a key pathological feature of OA. In advanced disease, chondrocyte loss leads to cell rarefaction, formation of empty lacunae, and progressive depletion of extracellular matrix components [16]. Previous studies have reported that the proportion of apoptotic chondrocytes in osteoarthritic cartilage can reach approximately 20% [17]. Accordingly, strategies aimed at inhibiting chondrocyte apoptosis have emerged as a promising direction for OA therapy.

HIF-2α is a transcription factor that regulates chondrocyte adaptation to hypoxic conditions and plays an essential role in maintaining cartilage homeostasis [18]. In the present study, we found that HIF-2α expression was significantly upregulated in osteoarthritic cartilage. Functionally, HIF-2α promoted chondrocyte apoptosis by suppressing HDAC4 expression and activating the ATF4/CHOP signaling pathway. Conversely, inhibition of HIF-2α reduced chondrocyte apoptosis, alleviated inflammatory responses, and exerted a protective effect on cartilage, suggesting a potential therapeutic target for OA by modulating chondrocyte survival.

Mechanistically, HIF-2α regulates downstream gene expression by sensing oxygen tension in chondrocytes and binding to hypoxia-responsive elements within target gene promoters and enhancers, thereby modulating transcriptional activity [19]. HDAC4 has been reported to suppress chondrocyte hypertrophy and apoptosis by inhibiting the ATF4/CHOP pathway [7,20]. Moreover, HDAC4 expression has been shown to decline in age-related spontaneous OA [20]. Although HIF-1 has been reported to inhibit HDAC4 transcriptional activity [21], the regulatory relationship between HIF-2α and HDAC4 has remained unclear. Our findings demonstrate that HIF-2α negatively regulates HDAC4 in OA, thereby activating the ATF4/CHOP axis and promoting chondrocyte apoptosis.

HIF-2α is widely recognized as a key transcriptional regulator in cartilage under hypoxic conditions and plays multiple roles in OA progression [22–25]. On one hand, HIF-2α directly induces the expression of catabolic enzymes and inflammatory mediators, including matrix metalloproteinases (MMP1, MMP3, MMP9, MMP12, and MMP13), ADAMTS4, inducible nitric oxide synthase (NOS2), and cyclooxygenase-2 (PTGS2), thereby contributing to extracellular matrix degradation [26–28]. On the other hand, HIF-2α has been implicated in promoting cell death through lipid peroxidation, reactive oxygen species (ROS) accumulation, and ferroptosis-related pathways, thereby sensitizing chondrocytes to degeneration [5,29]. In addition to these established mechanisms, our study identifies a novel apoptotic pathway regulated by HIF-2α in OA. Specifically, HIF-2α suppresses HDAC4 expression and activates the ATF4/CHOP signaling cascade, ultimately triggering chondrocyte apoptosis and accelerating disease progression. Furthermore, gait analysis revealed that inhibition of HIF-2α significantly improved pain-related behaviors in OA rats, suggesting functional benefits beyond structural protection. These findings expand the current understanding of HIF-2α in OA pathogenesis and provide experimental support for the potential therapeutic value of targeting HIF-2α in OA management.

However, several limitations of this study should be acknowledged. First, although we demonstrate a negative regulatory relationship between HIF-2α and HDAC4 in OA, the precise molecular mechanism underlying this interaction remains unclear. As a key transcription factor, HIF-2α typically activates gene expression by binding to hypoxia-response elements. Nevertheless, accumulating evidence suggests that members of the HIF family may also function as transcriptional repressors. For example, HIF-1α has been reported to inhibit the expression or activity of specific histone deacetylases to maintain cellular homeostasis [21]. In OA, HIF-2α may suppress HDAC4 expression either directly, through recruitment of transcriptional co-repressors to its promoter, or indirectly via regulation of intermediary non-coding RNAs. Notably, previous studies have shown that the long non-coding RNA HDAC4-AS1 can significantly downregulate HDAC4 expression under hypoxic conditions, providing a potential mechanistic framework for our findings. However, due to the absence of dual-luciferase reporter and chromatin immunoprecipitation (ChIP) assays in the present study, whether HIF-2α directly targets the HDAC4 promoter remains to be determined and will be the focus of future work. Second, OA is a complex joint disease involving multiple cell types, including fibroblasts, osteoblasts, osteoclasts, and synoviocytes. The role of HIF-2α in these cellular populations has not been investigated in the present study and warrants further exploration. Finally, while the ACLT model is widely used to mimic OA, it does not fully recapitulate the clinical condition, which often occurs in aged individuals with comorbidities. Future studies using more clinically relevant models, such as HIF-2α conditional knockout mice, may provide deeper insight into its role in OA pathogenesis.

In conclusion, our findings indicate that HIF-2α promotes chondrocyte apoptosis and cartilage degeneration in OA by suppressing HDAC4 expression and activating the ATF4/CHOP signaling pathway. This study identifies a novel regulatory mechanism of HIF-2α in OA and provides new insights that may inform the development of targeted therapeutic strategies for this disease.

## Supporting information

S1 File Supplemental files.‌‌ (DOCX)

S2 File Raw images. (PDF)
