# Supplementary material for: HIF-2α drives osteoarthritis progression via suppression of the HDAC4-ATF4-CHOP signaling axis
Source: PLoS One. 2026 Jun 18;21(6):e0351847. doi: 10.1371/journal.pone.0351847 (PMC13278430; doi:10.1371/journal.pone.0351847)
Supplement: S1 File — (DOCX) [file pone.0351847.s001.docx]

**HIF-2α promotes osteoarthritis progression by inhibiting the HDAC4/ATF4/CHOP signalling pathway**

Pinpin Jiang ^1,¶^, Hang Wang ^1,¶^, Yujia Li ^1,2^, Yuanyu Zhang ^1^ , Jingrui Huang ^1^, Yukun Xu^1^, Dahai Rong ^1^, Danni Ruan ^1,2^, Yao Wang ^1,2^, Jie Yuan ^1^, Pengcui Li ^1*^

^1^  Key Laboratory of Bone and Soft Tissue Injury, Second Hospital of Shanxi Medical University, Taiyuan,China

^2^ Academy of Medical Sciences, Shanxi Medical University, Taiyuan, China

^¶^Authors made equal contributions to the manuscript and are co-first authors

**^*^**Correspondence author

E-mail: lpc1977@163.com

Supplementary table 1:Detailed information and clinical data of the human cartilage donors.

| Diagnosis | ICRS Grade |
| --- | --- |
| Right tibial plateau fracture | 0 |
| Right tibial plateau fracture | 0 |
| Right tibial plateau fracture | 0 |
| OA | 3 |
| OA | 4 |
| OA | 4 |

Supplementary Table 2. Sequences of primer pairs used for RT-qPCR in this study

|  | Gene | Sequences |
| --- | --- | --- |
| human | HIF-2α | F:5'-GTGACATGATCTTTCTGTCGGAA-3' |
|  |  | R:5'-CGCAAGGATGAGTGAAGTCAAA-3' |
|  | HDAC4 | F:5'-GCCAAAGATGACTTCCCTCTTA-3' |
|  |  | R:5'-TTTCGGCCACTTTCTGCTTTAG-3' |
|  | ATF4 | F:5'-ATGGATTTGAAGGAGTTCGACT-3' |
|  |  | R:5'-AGAGATCACAAGTGTCATCCAA-3' |
|  | CHOP | F:5'-GAGAATGAAAGGAAAGTGGCAC-3' |
|  |  | R:5'-ATTCACCATTCGGTCAATCAGA-3' |
|  | Caspase3 | F:5'-GGAACCAAAGATCATACATGG-3' |
|  |  | R:5'-AGTTTCTGAATGTTTCCCTG-3' |
|  | Gene | Sequences |
| Rat | HIF-2α | F:5'-CCTGATTGTGCGGGACTCTC-3' |
|  |  | R:5'-CTCCGAGCTGCTCCTTTTCT-3' |
|  | HDAC4 | F:5ʹ-GGCTTCCTTGTGGTGGTGTTGG-3ʹ |
|  |  | R:5ʹ-TGTACTCTCCTCGGCATGGTGTC-3ʹ |
|  | ATF4 | F:5ʹ-GACCGAGATGAGCTTCCTGAACAG-3ʹ |
|  |  | R:5ʹ-CCGCCTTGTCGCTGGAGAAC-3ʹ |
|  | CHOP | F:5ʹ-CCTCGCTCTCCAGATTCCAGTCAG-3ʹ |
|  |  | R:5ʹ-TCTCCTGCTCCTTCTCCTTCATGC-3ʹ |
|  | Caspase3 | F:5ʹ-ATGGAGAACAATAAAACCT -3ʹ |
|  |  | R:5ʹ-CTAGTGATAAAAGTAGAGTTC-3ʹ |
|  | Caspase9 | F:5'- GCTGAGATGACGAGCACCTT -3' |
|  |  | R:5'- GCTGGTTTGAGGTTGAGGAA -3' |
|  | COL10 | F:5ʹ-GGATGCCTCTTGTCAGTGCTAACC-3ʹ |
|  |  | R:5ʹ-TCATAGTGCTGCTGCCTGTTGTAC-3ʹ |
|  | MMP13 | F:5ʹ-ATACGAGCATCCATCCCGAGACC-3ʹ |
|  |  | R:5ʹ-AACCGCAGCACTGAGCCTTTTC-3ʹ |

Supplementary figure 1:


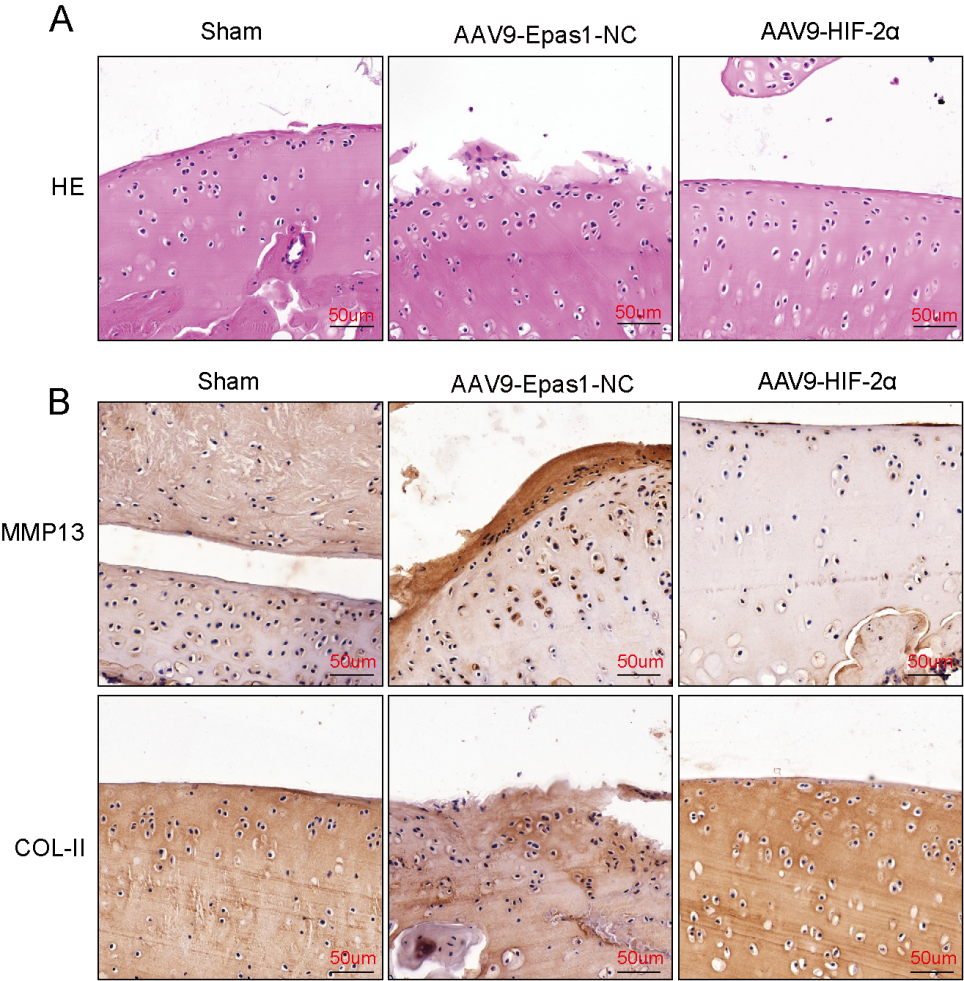


Figure S1: Inhibition of HIF-2α can alleviate the progression of osteoarthritis. A: Representative H&E staining images for the assessment of articular surface damage (n = 3), Scale bar = 50um; B: Representative images of immunohistochemical staining for MMP13, COL-Ⅱ in the three groups of rats at 12 weeks (n = 3), Scale bar = 50um.
