# Supplementary material for: HIF-2α drives osteoarthritis progression via suppression of the HDAC4-ATF4-CHOP signaling axis
Source: PLoS One. 2026 Jun 18;21(6):e0351847. doi: 10.1371/journal.pone.0351847 (PMC13278430; doi:10.1371/journal.pone.0351847)
Supplement: S2 File — (PDF) [file pone.0351847.s002.pdf]

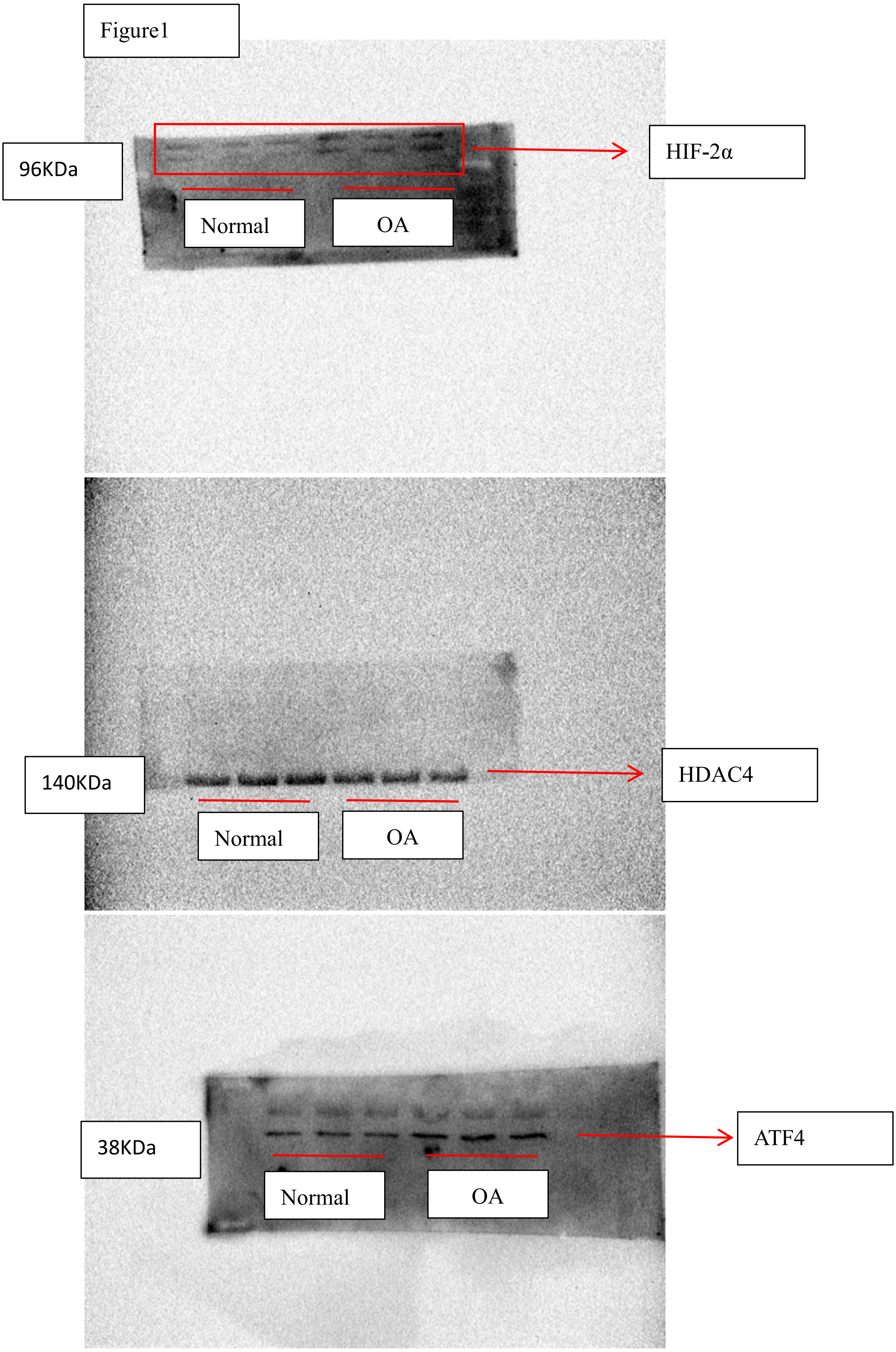
Figure1
96KDa
HIF-2α
Normal
OA
140KDa
HDAC4
Normal
OA
38KDa
ATF4
Normal
OA

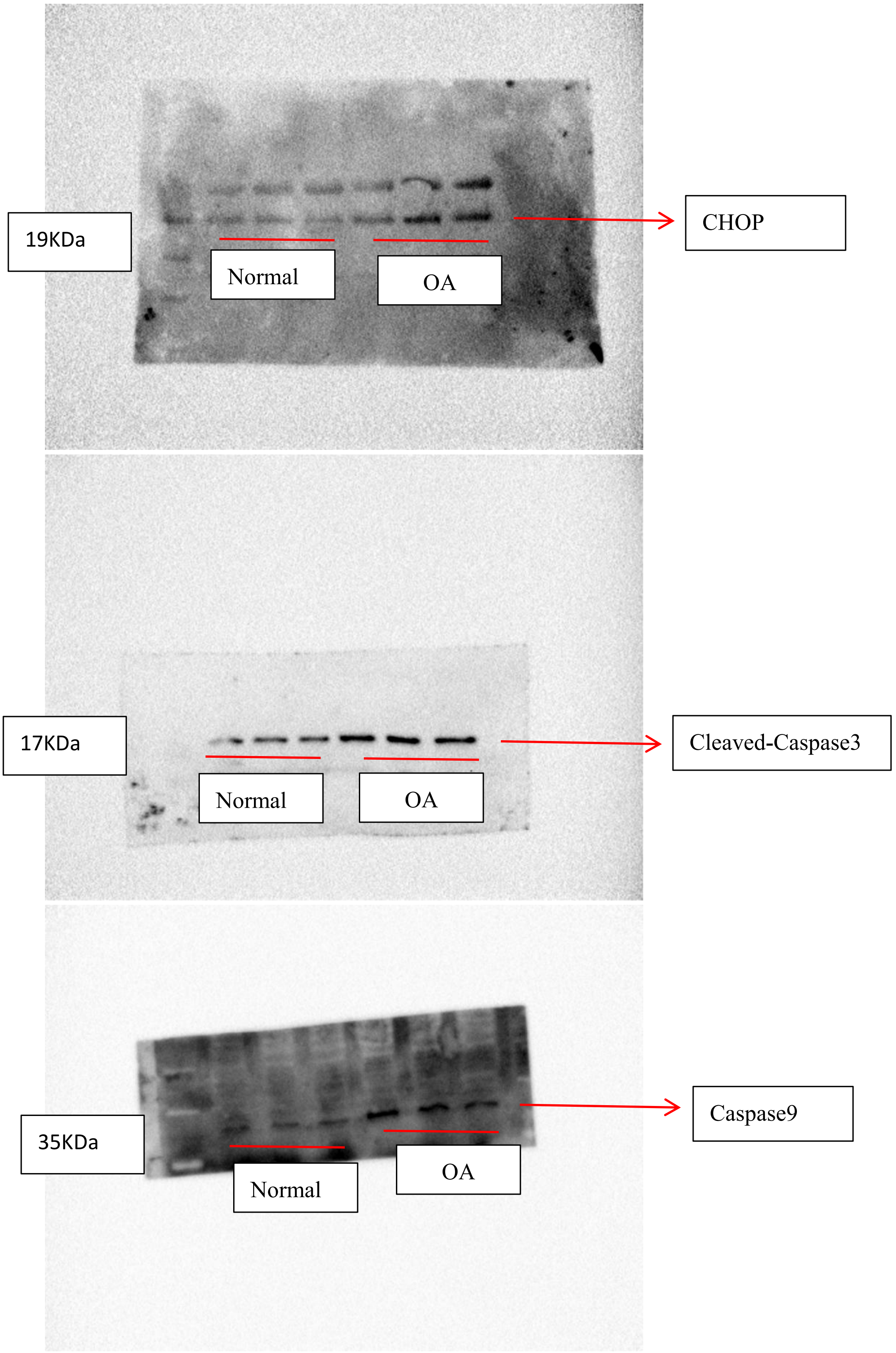
19KDa
Normal
OA
CHOP
17KDa
Normal
OA
Cleaved-Caspase3
35KDa
Normal
OA
Caspase9

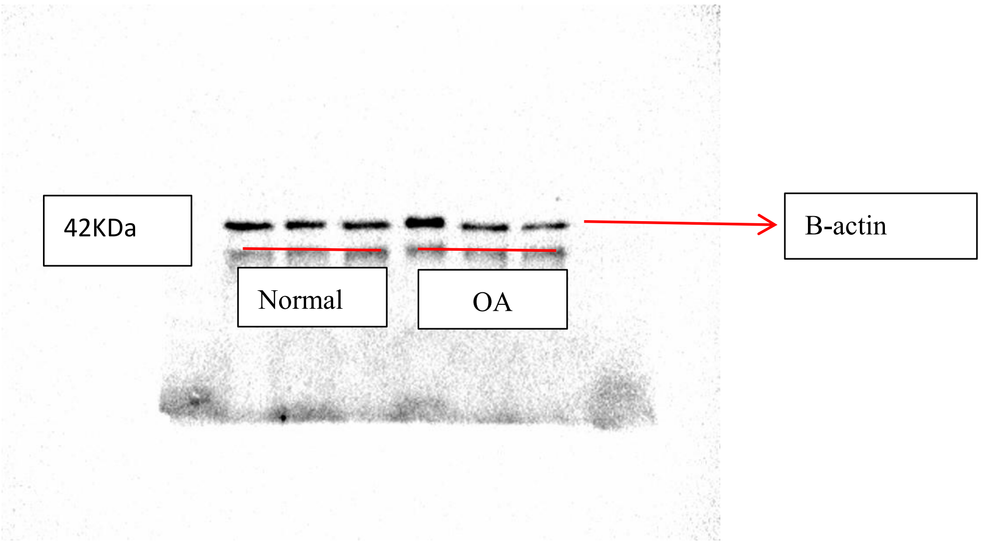
42KDa
B-actin
Normal
OA

Figure2,3

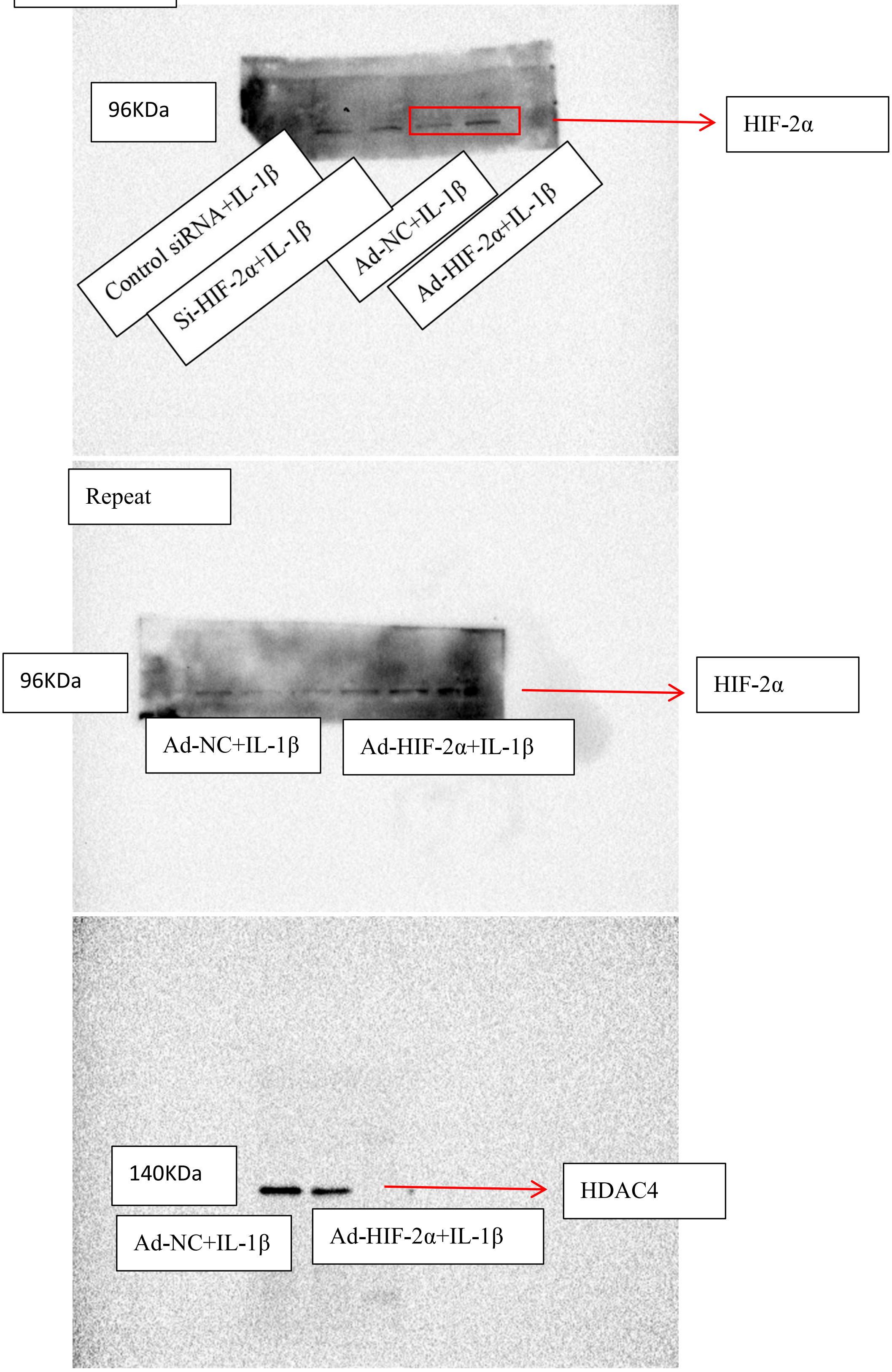
96KDa
Control siRNA+IL-1β
Si-HIF-2α+IL-1β
Ad-NC+IL-1β
Ad-HIF-2α+IL-1β
HIF-2α
Repeat
96KDa
Ad-NC+IL-1β
Ad-HIF-2α+IL-1β
HIF-2α
140KDa
Ad-NC+IL-1β
Ad-HIF-2α+IL-1β
HDAC4

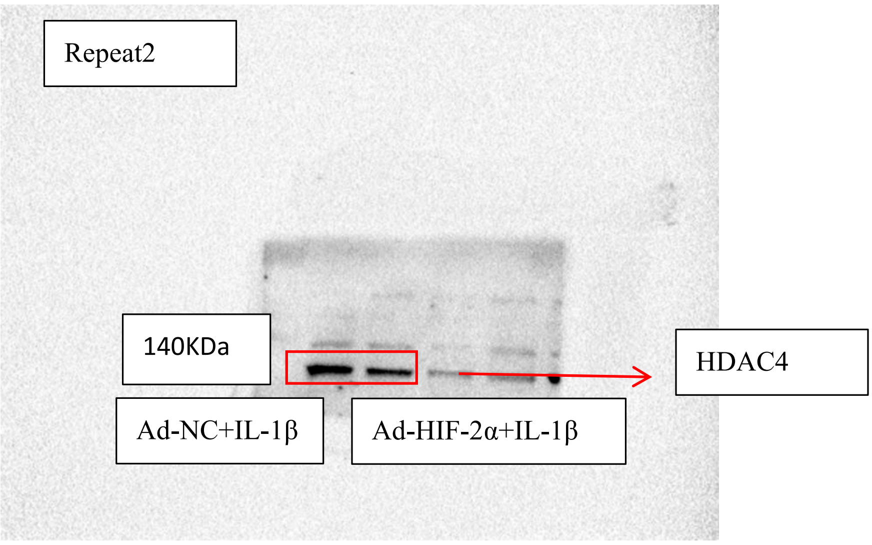
Repeat2
140KDa
HDAC4
Ad-NC+IL-1β
Ad-HIF-2α+IL-1β

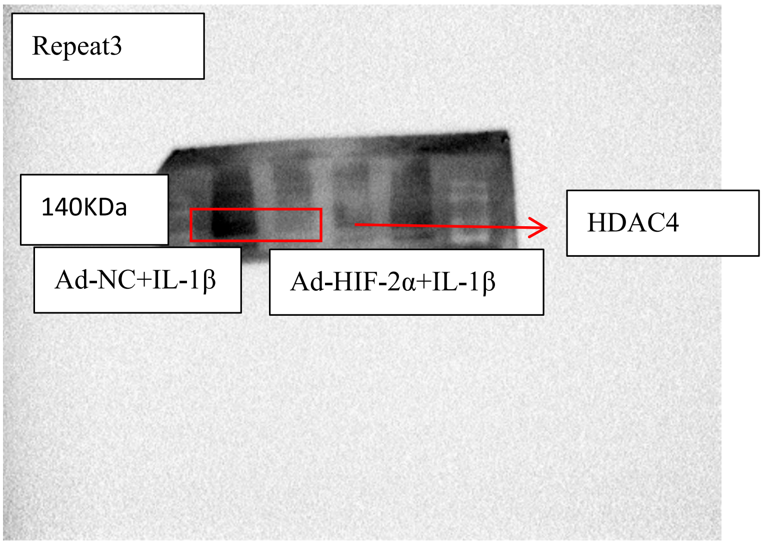
Repeat3
140KDa
HDAC4
Ad-NC+IL-1β
Ad-HIF-2α+IL-1β

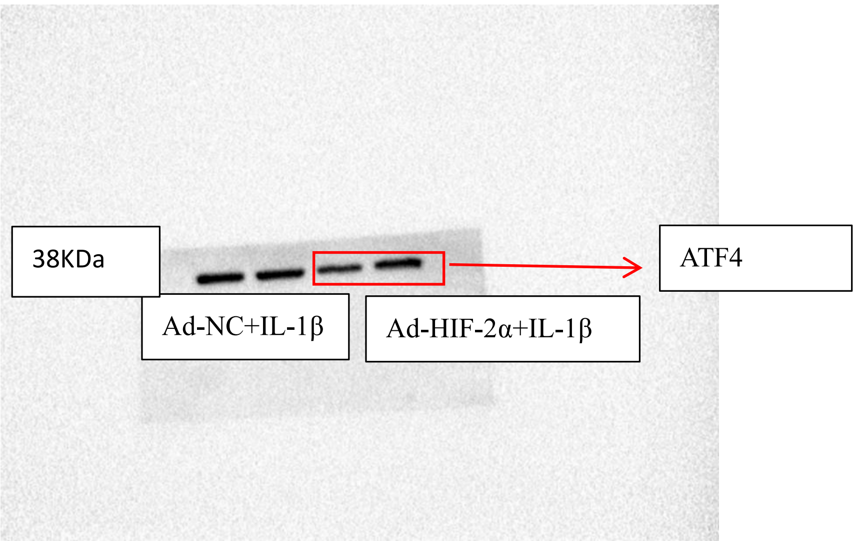
38KDa
ATF4
Ad-NC+IL-1β
Ad-HIF-2α+IL-1β

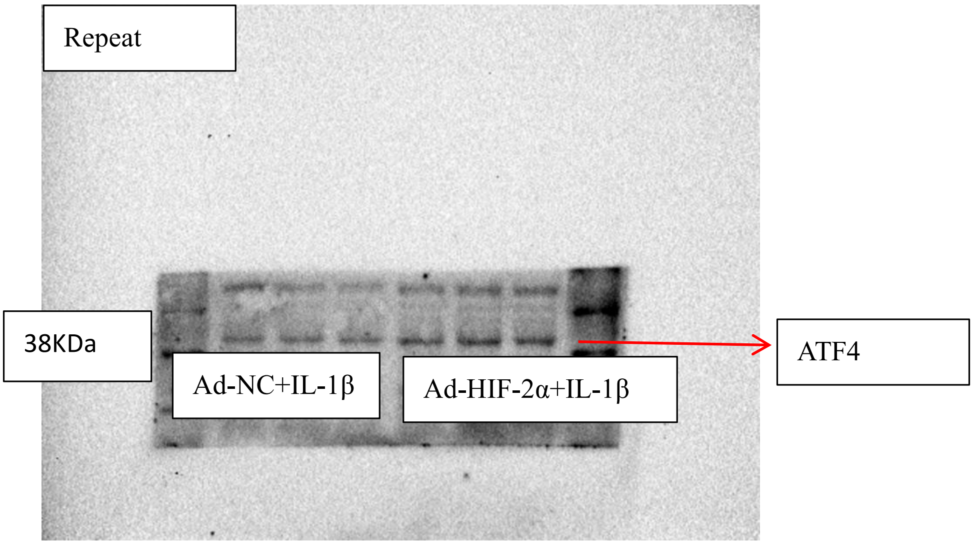
Repeat
38KDa
Ad-NC+IL-1β
Ad-HIF-2α+IL-1β
ATF4

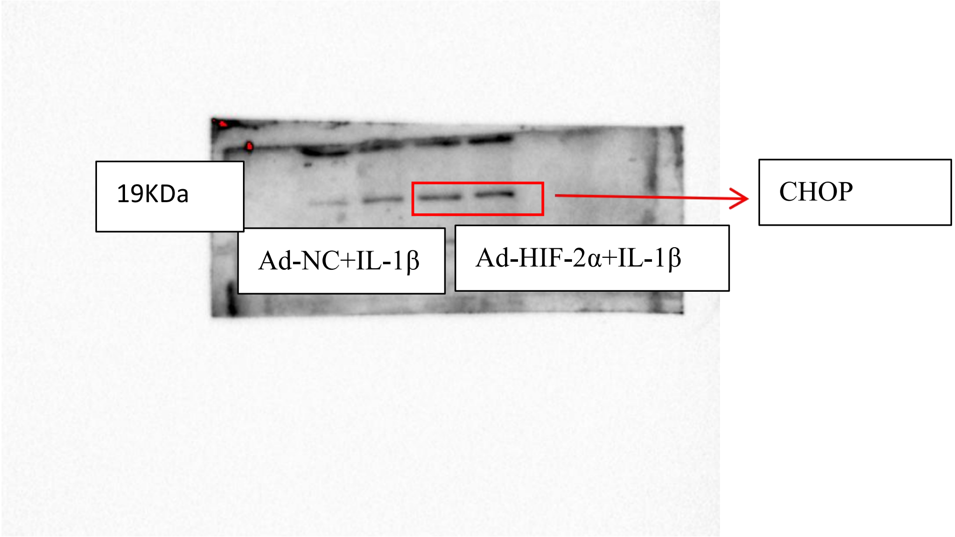
19KDa
Ad-NC+IL-1β
Ad-HIF-2α+IL-1β
CHOP

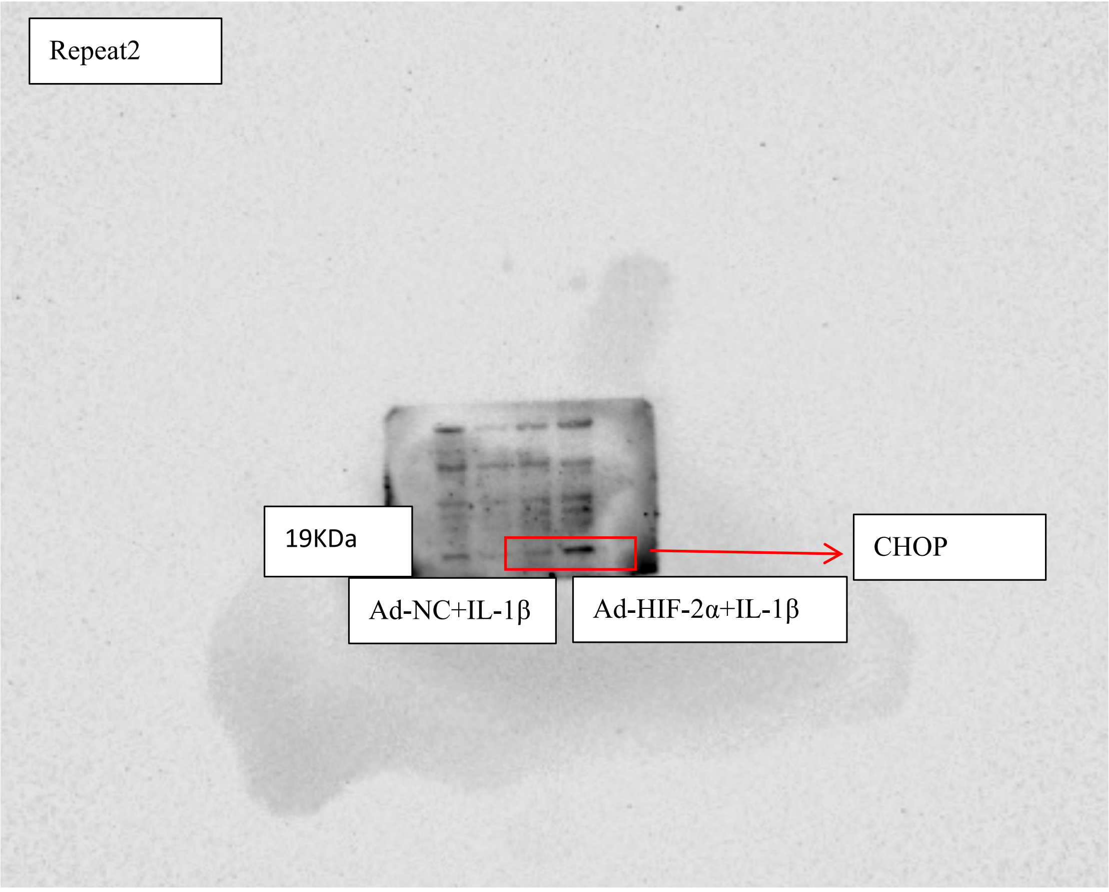
Repeat2
19KDa
CHOP
Ad-NC+IL-1β
Ad-HIF-2α+IL-1β

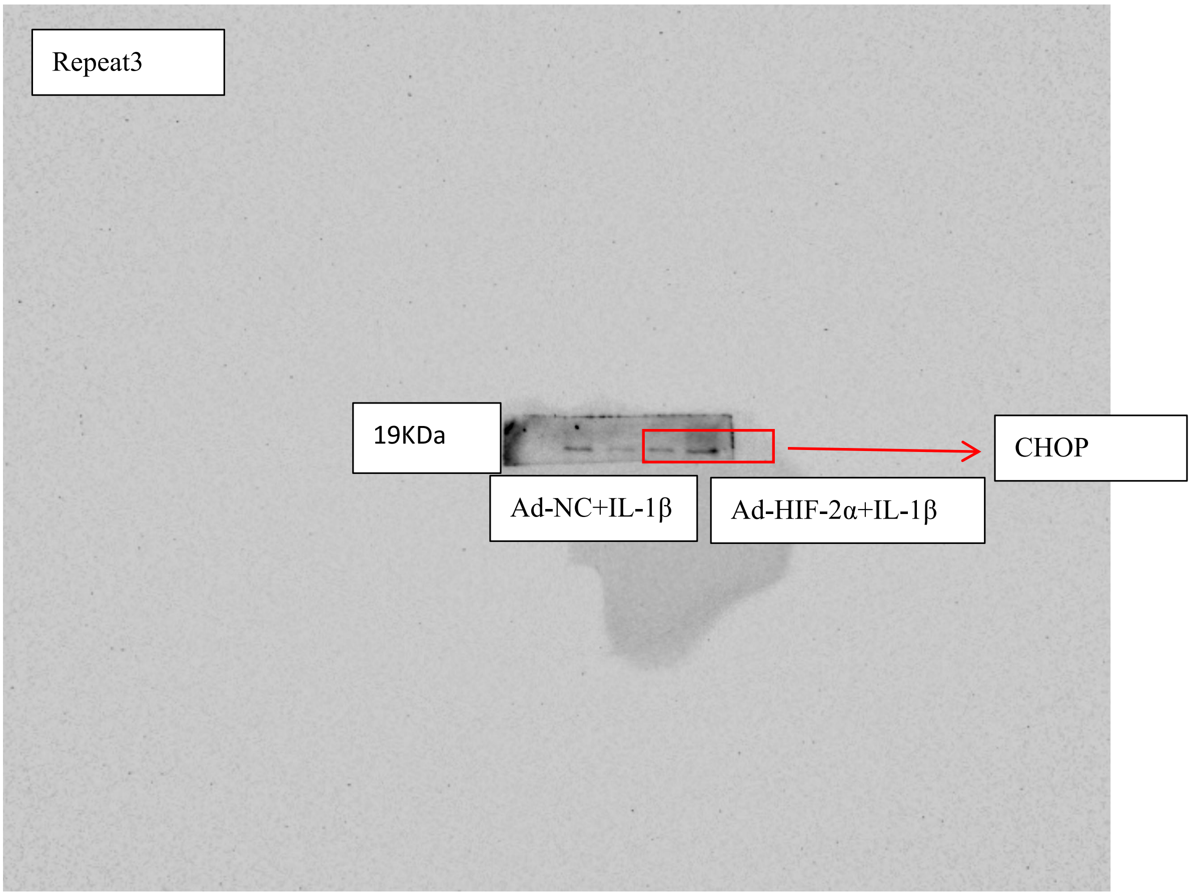
Repeat3
19KDa
CHOP
Ad-NC+IL-1β
Ad-HIF-2α+IL-1β

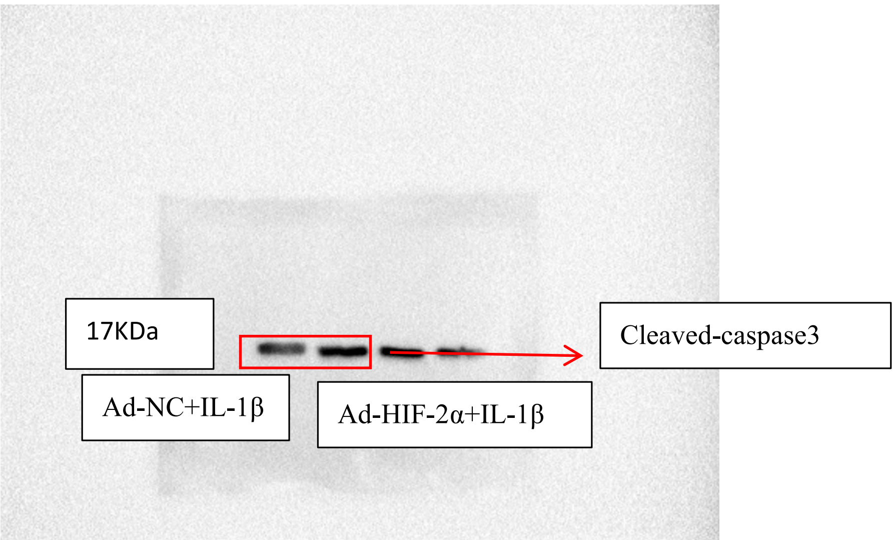
17KDa
Cleaved-caspase3
Ad-NC+IL-1β
Ad-HIF-2α+IL-1β

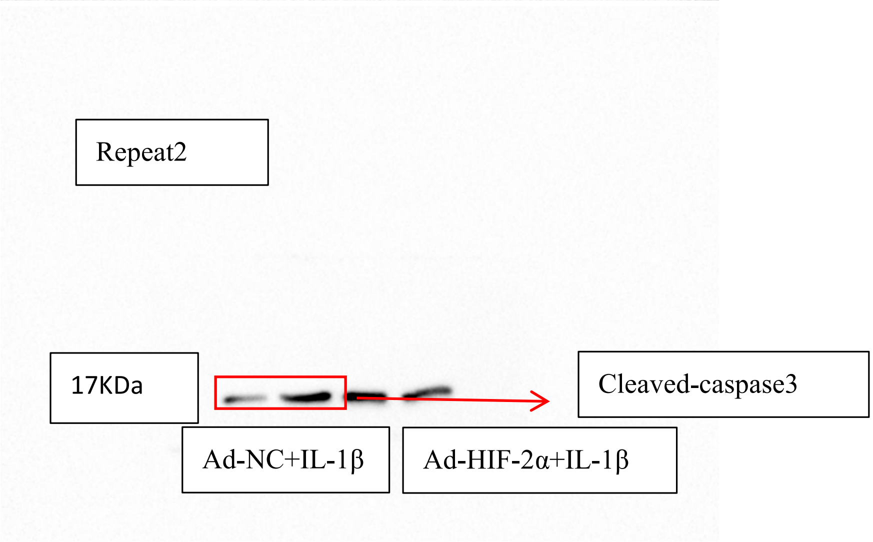
Repeat2
17KDa
Cleaved-caspase3
Ad-NC+IL-1β
Ad-HIF-2α+IL-1β

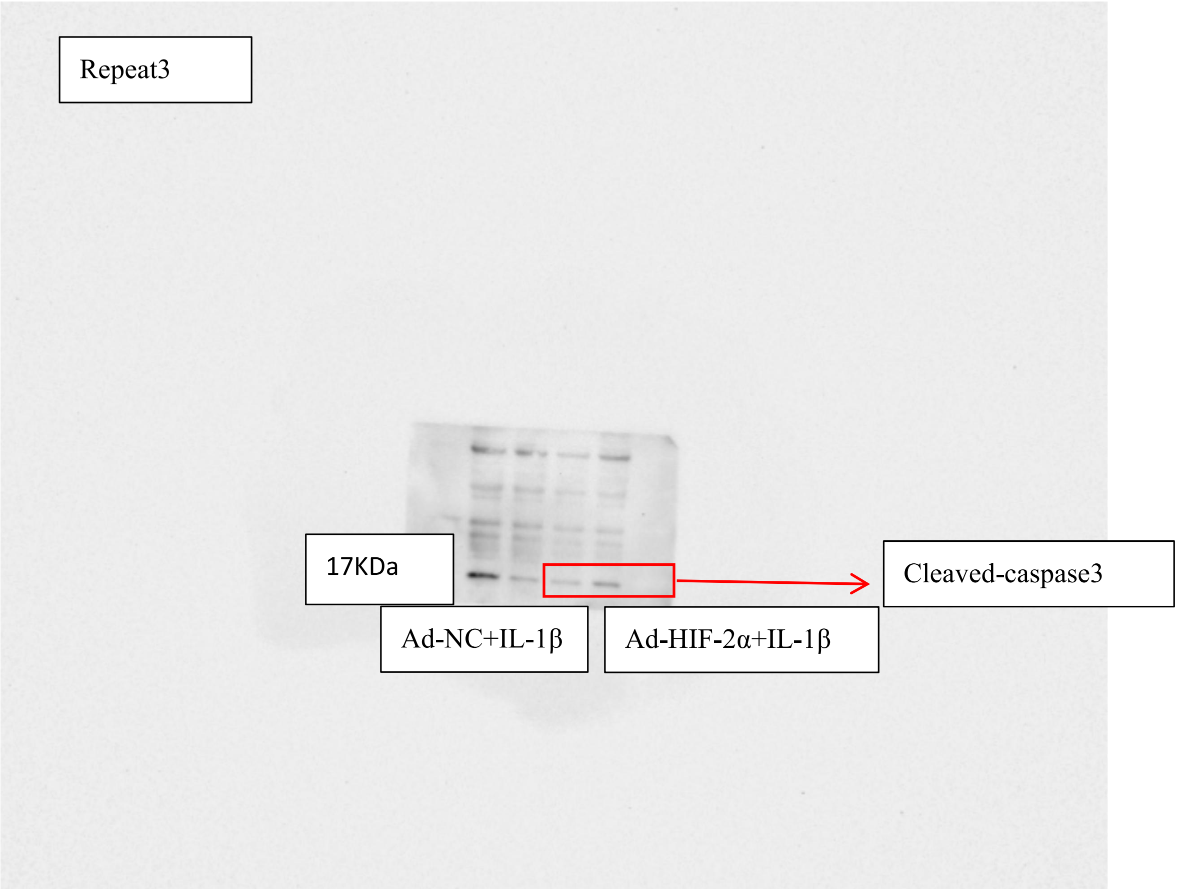
Repeat3
17KDa
Cleaved-caspase3
Ad-NC+IL-1β
Ad-HIF-2α+IL-1β

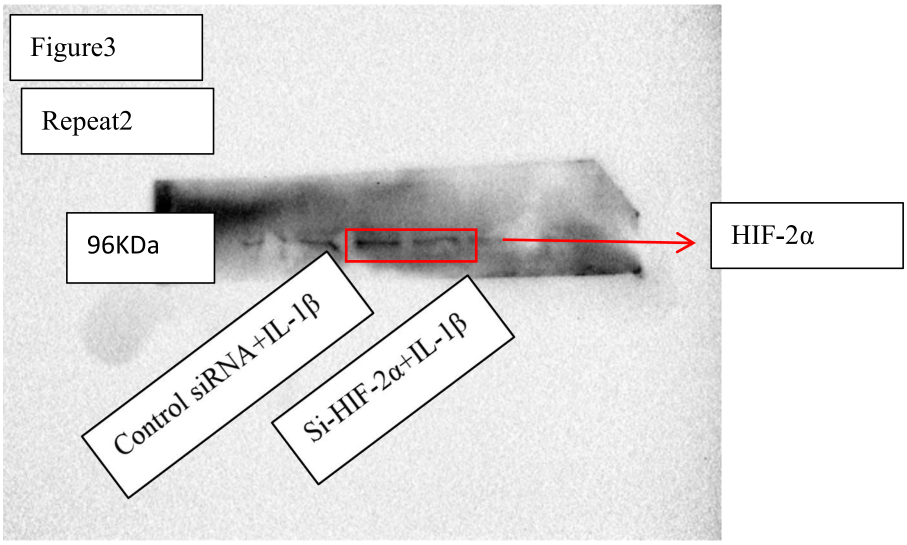
Figure3
Repeat2
96KDa
HIF-2α
Control siRNA+IL-1β
Si-HIF-2α+IL-1β

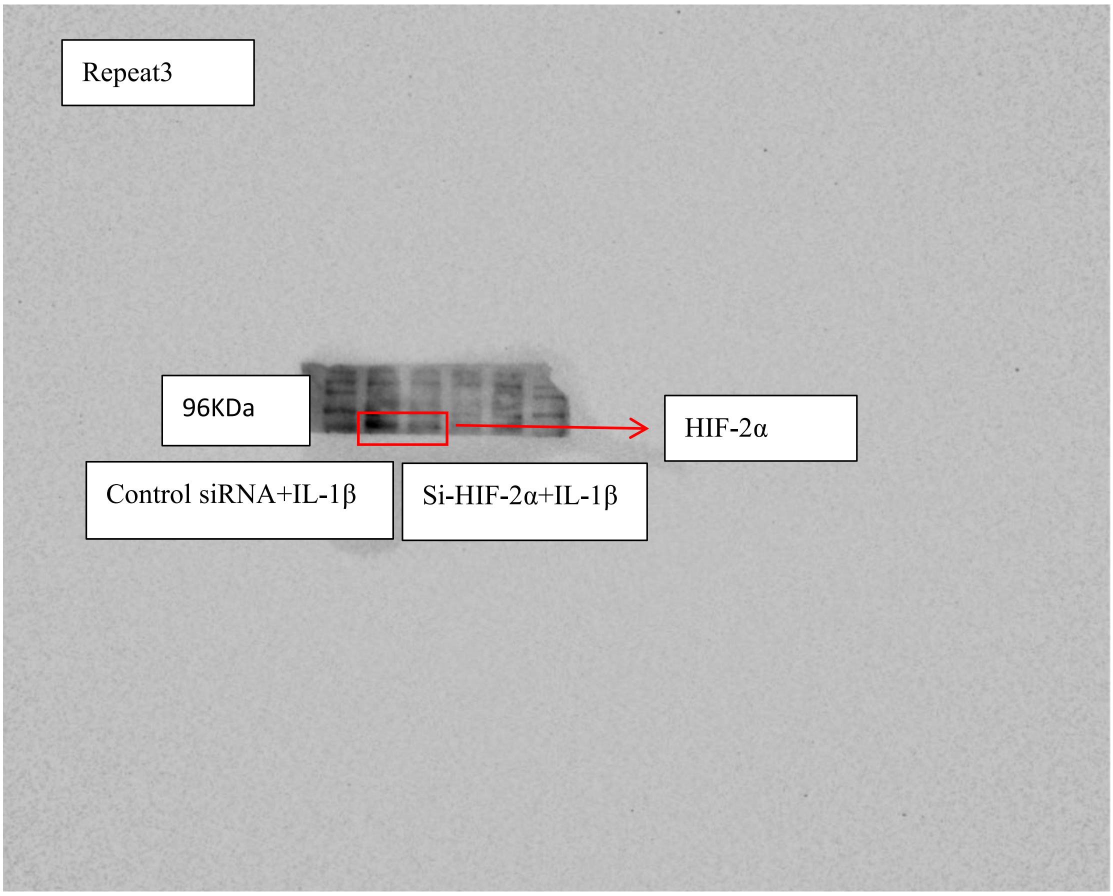
Repeat3
96KDa
HIF-2α
Control siRNA+IL-1β
Si-HIF-2α+IL-1β

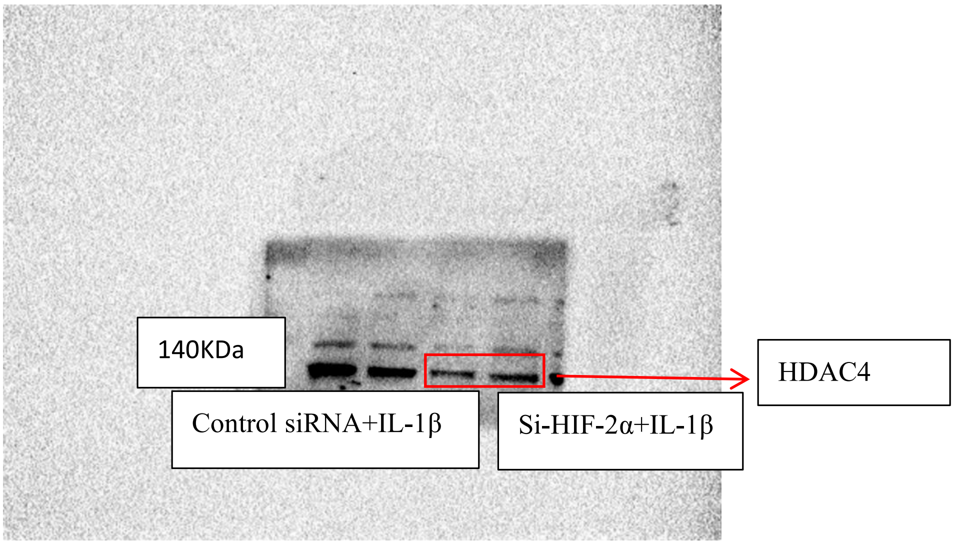
140KDa
HDAC4
Control siRNA+IL-1β
Si-HIF-2α+IL-1β

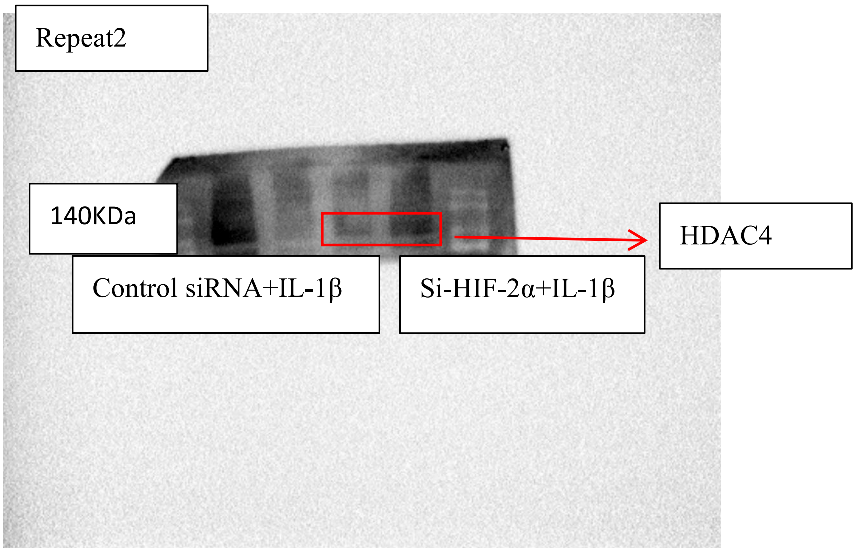
Repeat2
140KDa
HDAC4
Control siRNA+IL-1β
Si-HIF-2α+IL-1β

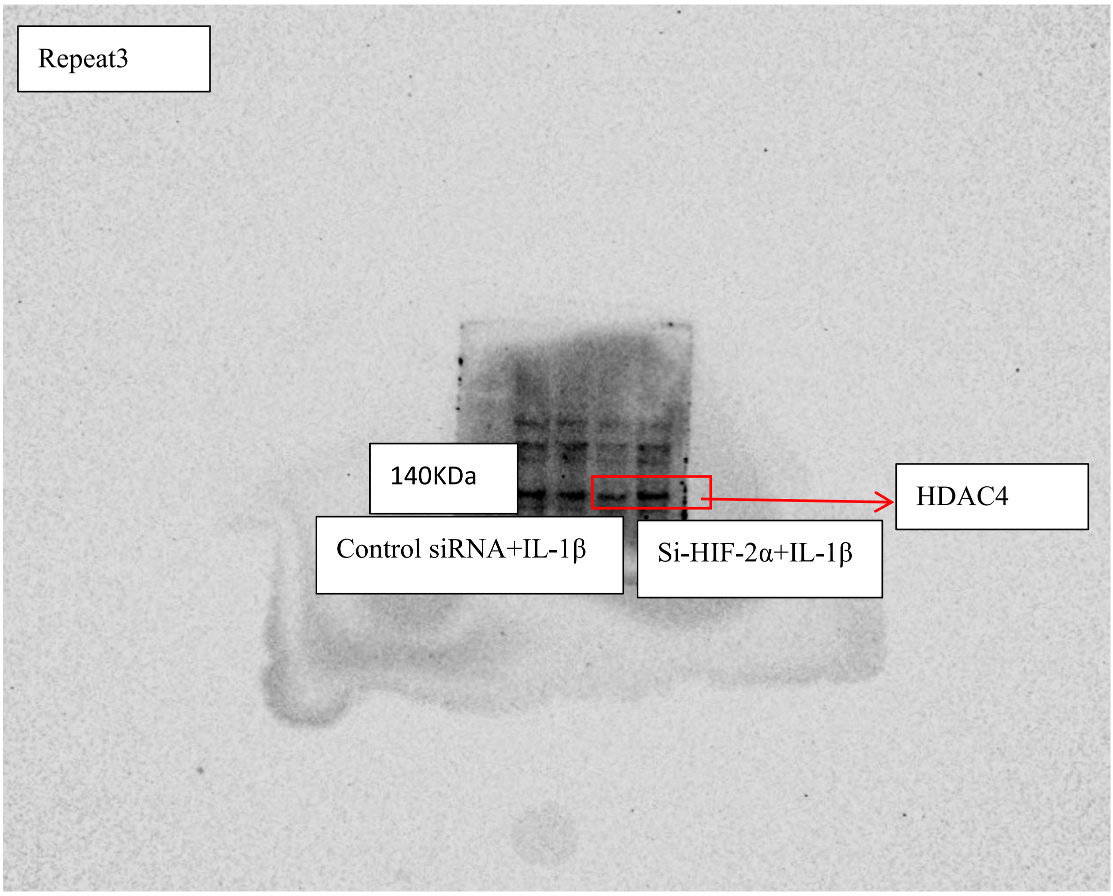
Repeat3
140KDa
HDAC4
Control siRNA+IL-1β
Si-HIF-2α+IL-1β

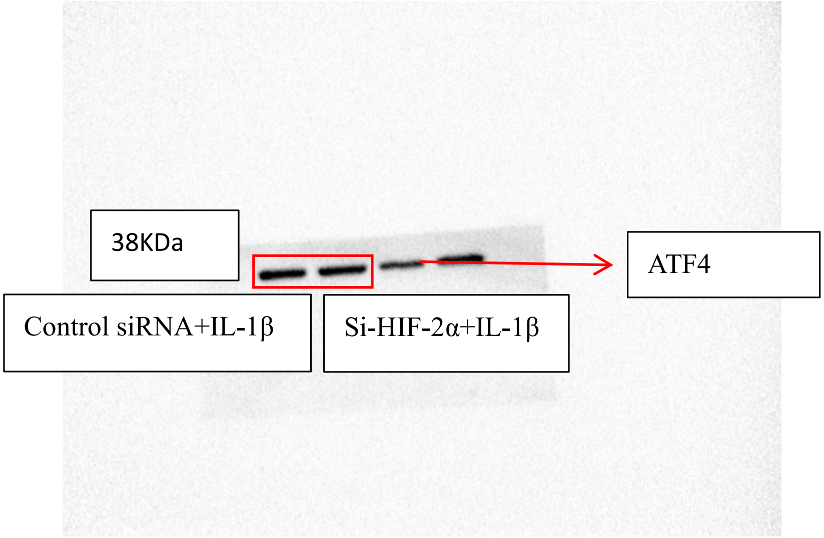
38KDa
ATF4
Control siRNA+IL-1β
Si-HIF-2α+IL-1β

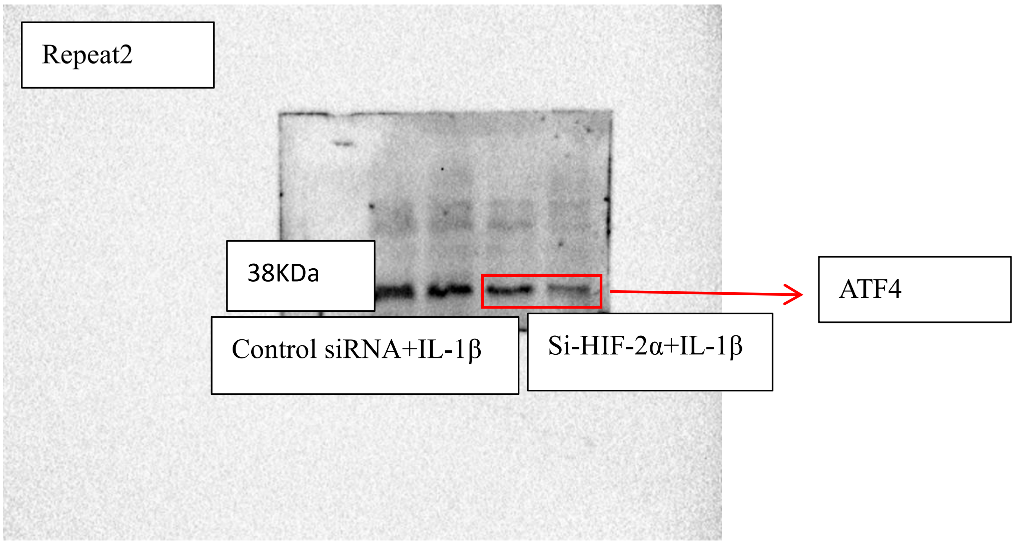
Repeat2
38KDa
ATF4
Control siRNA+IL-1β
Si-HIF-2α+IL-1β

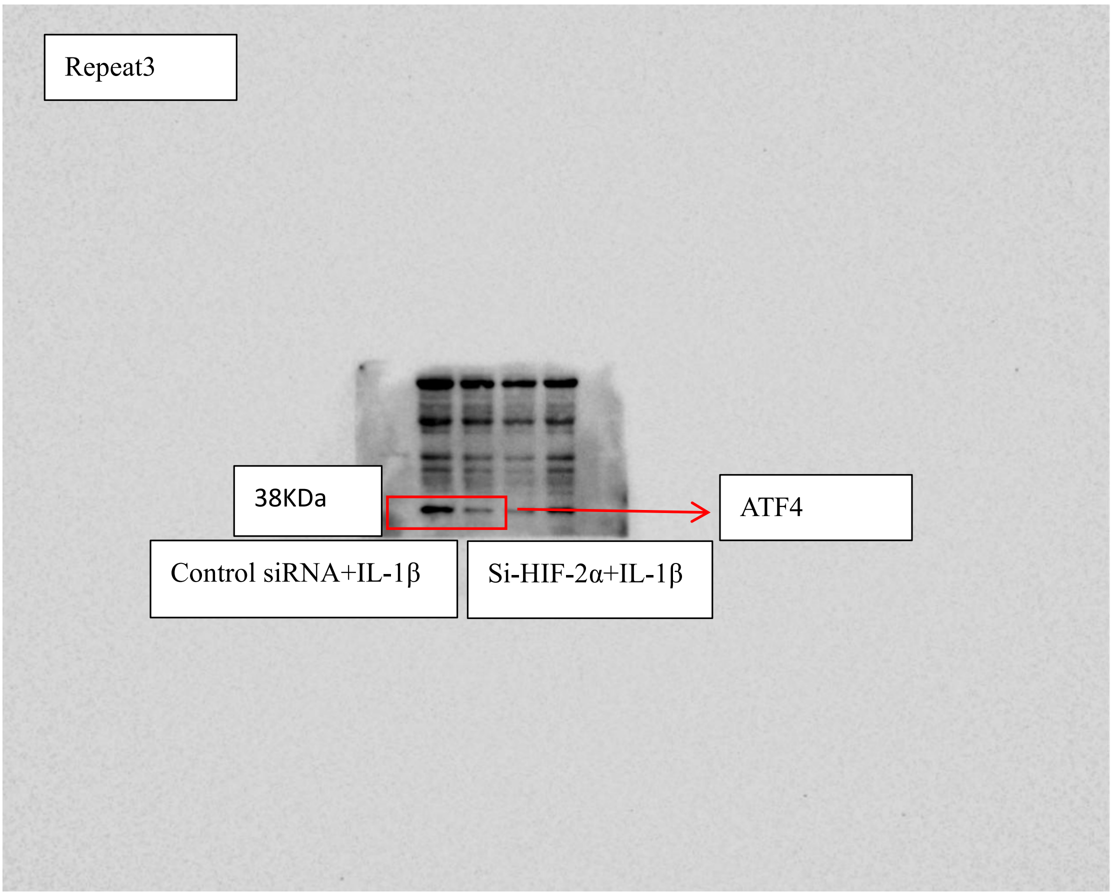
Repeat3
38KDa
ATF4
Control siRNA+IL-1β
Si-HIF-2α+IL-1β

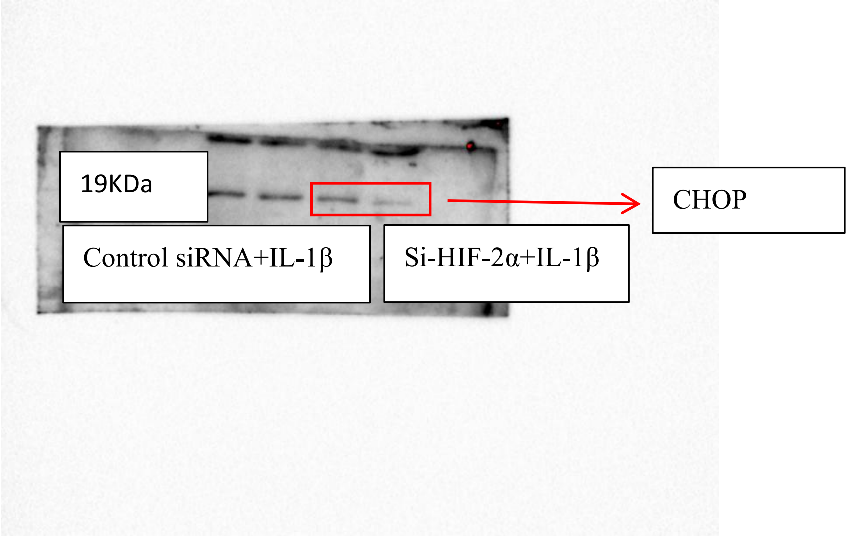
19KDa
CHOP
Control siRNA+IL-1β
Si-HIF-2α+IL-1β

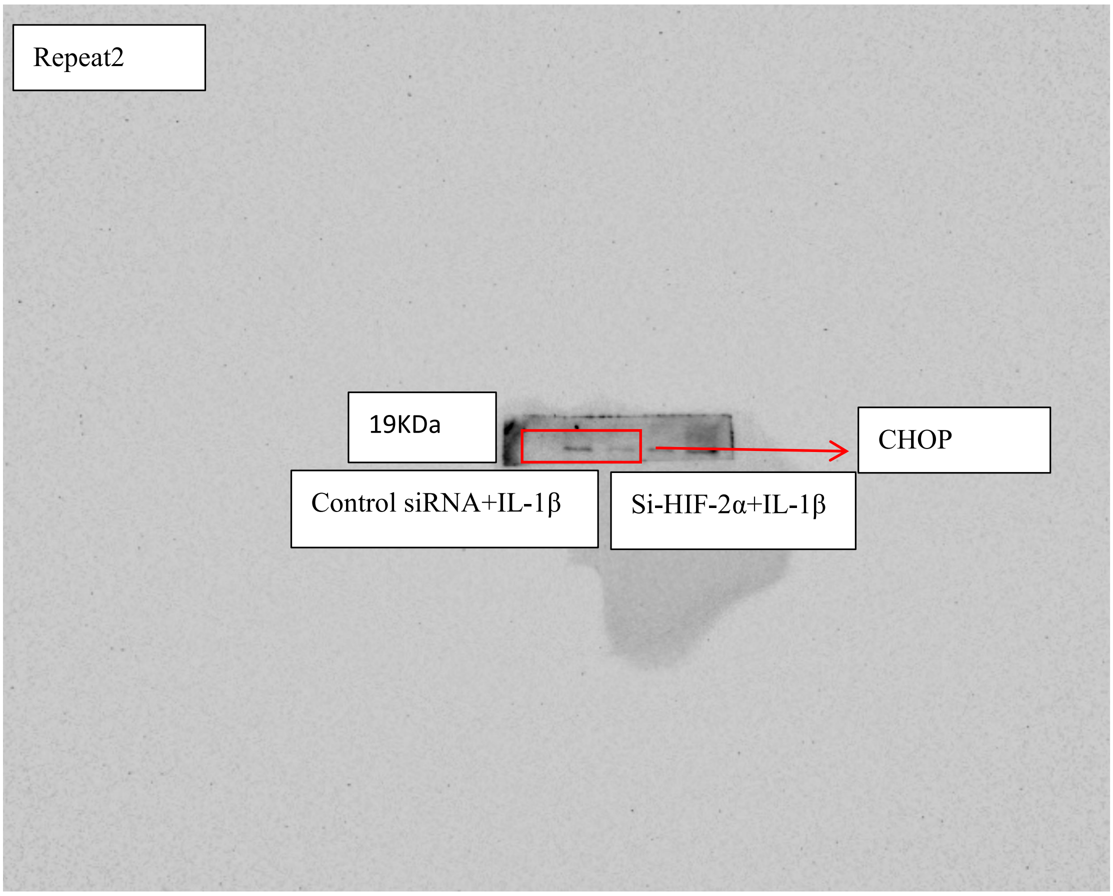
Repeat2
19KDa
CHOP
Control siRNA+IL-1β
Si-HIF-2α+IL-1β

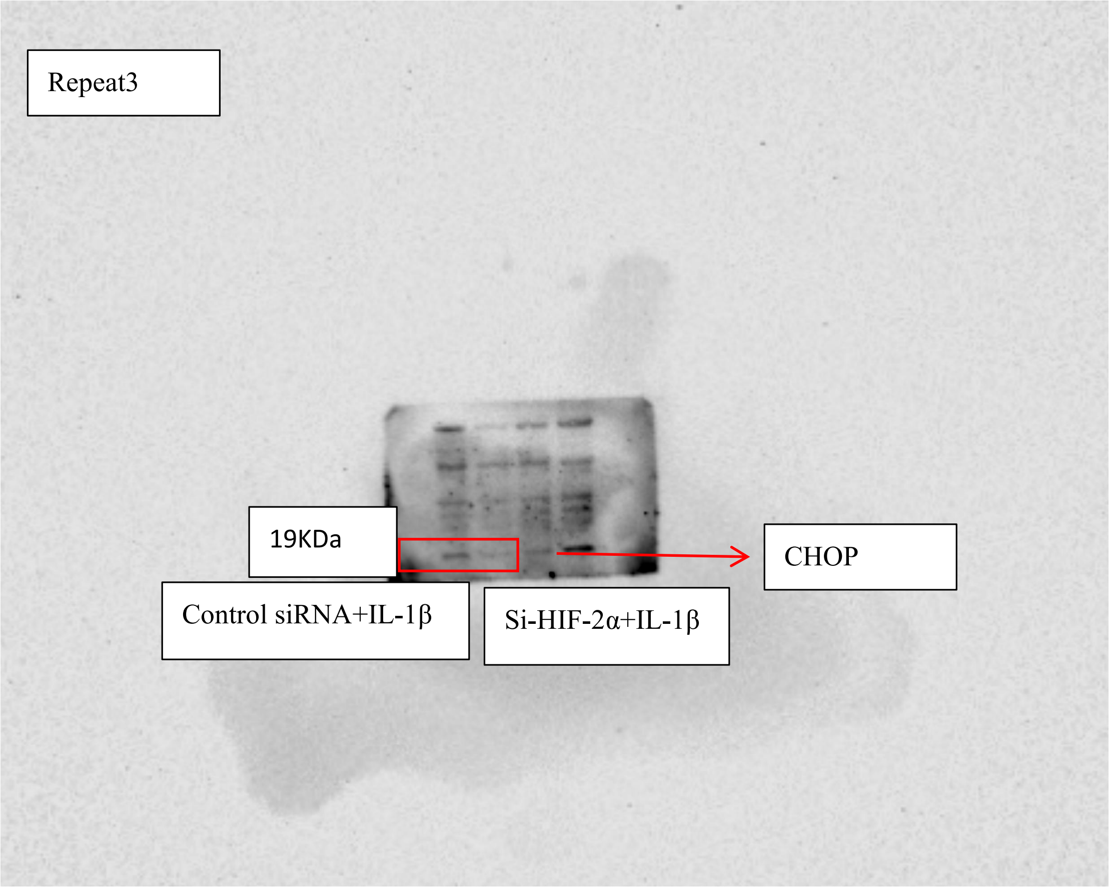
Repeat3
19KDa
CHOP
Control siRNA+IL-1β
Si-HIF-2α+IL-1β

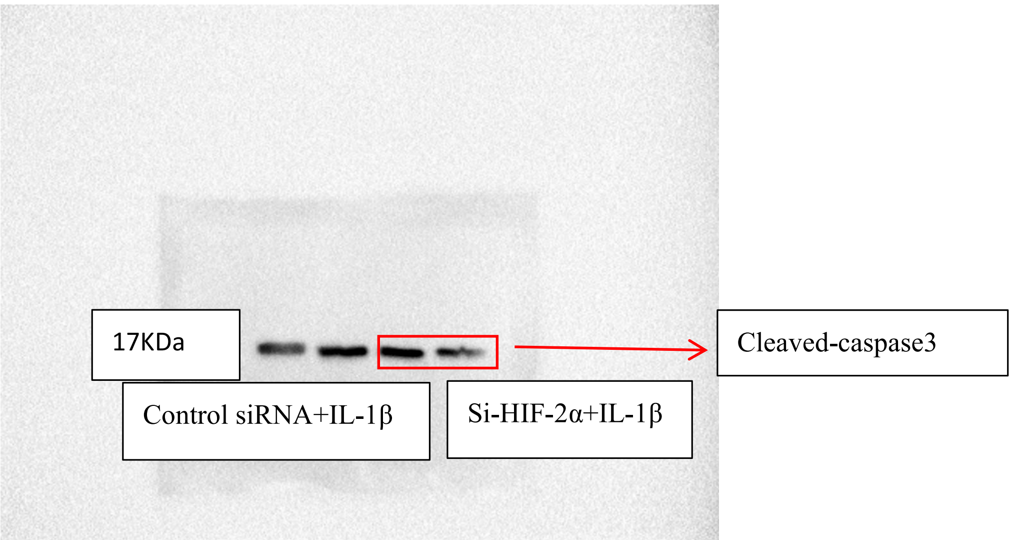
17KDa
Control siRNA+IL-1β
Si-HIF-2α+IL-1β
Cleaved-caspase3

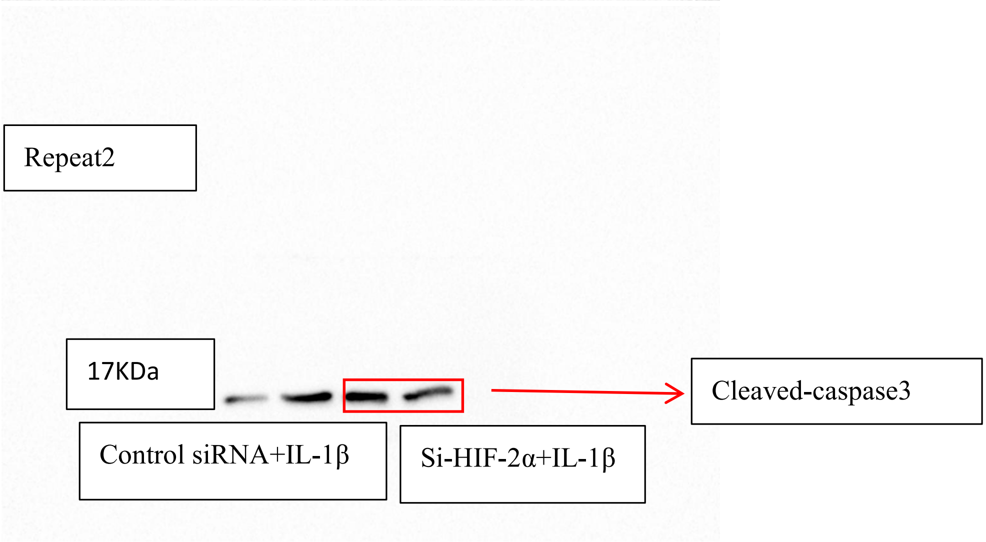
Repeat2
17KDa
Control siRNA+IL-1β
Si-HIF-2α+IL-1β
Cleaved-caspase3

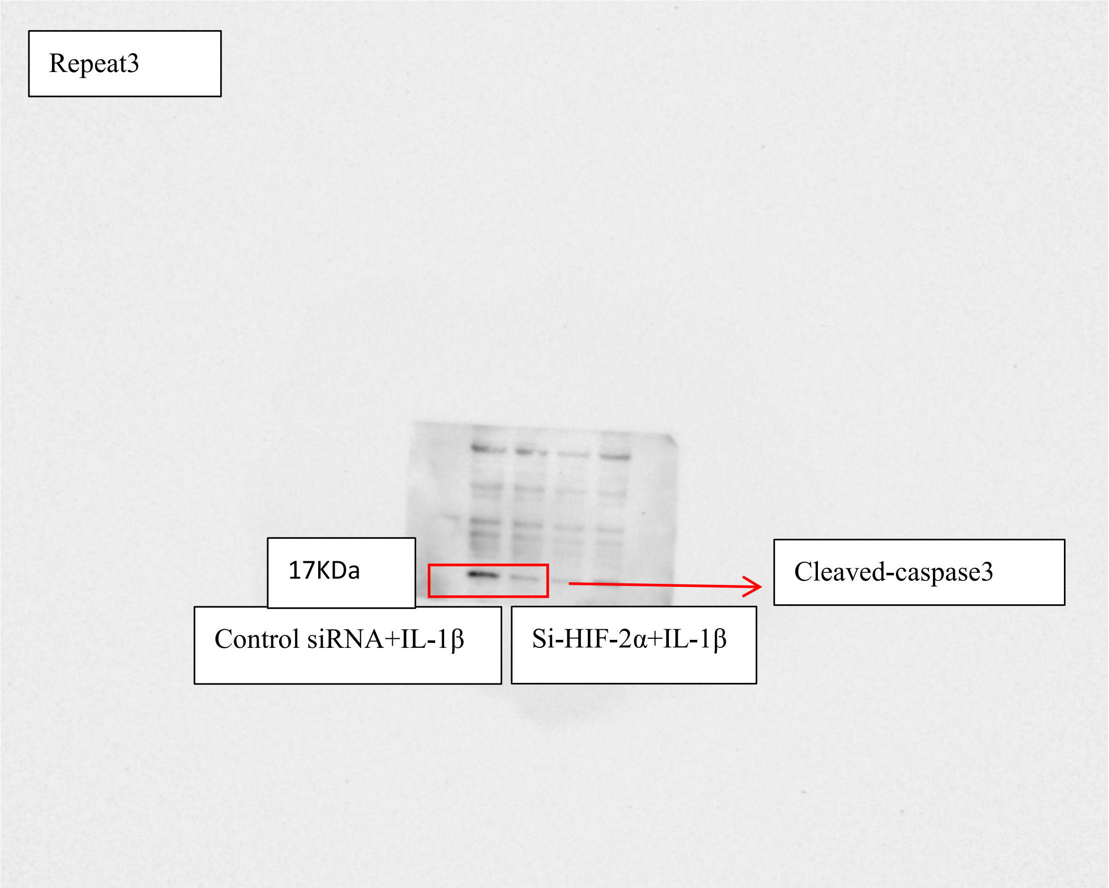
Repeat3
17KDa
Cleaved-caspase3
Control siRNA+IL-1β
Si-HIF-2α+IL-1β

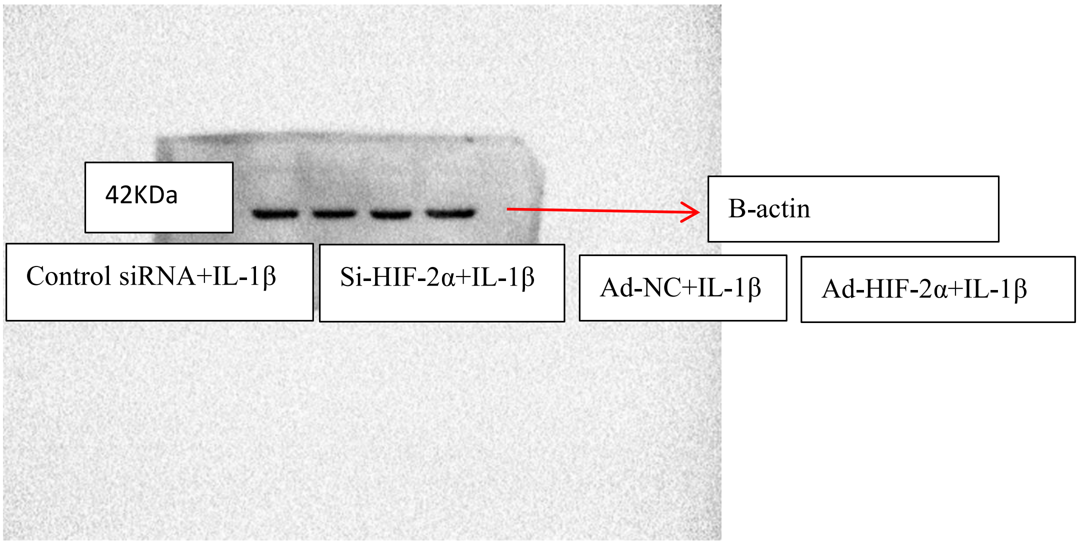
42KDa
B-actin
Control siRNA+IL-1β
Si-HIF-2α+IL-1β
Ad-NC+IL-1β
Ad-HIF-2α+IL-1β

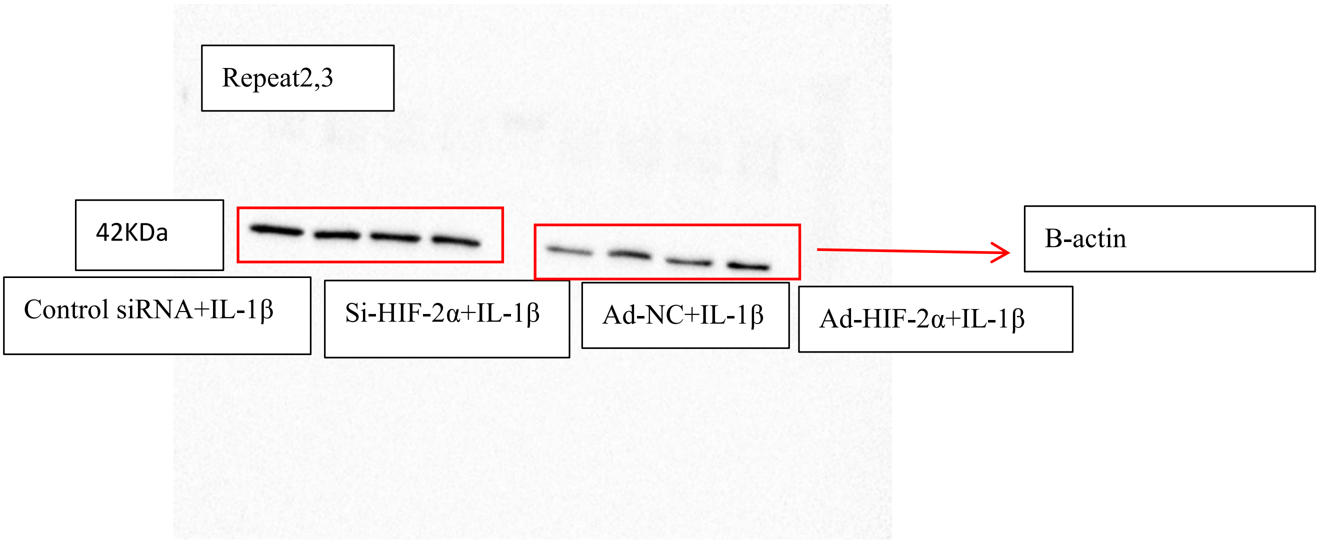
Repeat2,3
42KDa
B-actin
Control siRNA+IL-1β
Si-HIF-2α+IL-1β
Ad-NC+IL-1β
Ad-HIF-2α+IL-1β
